# The effects of developmental cadmium exposure on health and disease

**DOI:** 10.1242/dmm.052038

**Published:** 2025-06-09

**Authors:** Kathleen M. Hudson, Logan Dameris, Rebecca Lichtler, Michael Cowley

**Affiliations:** ^1^ Department of Biological Sciences, North Carolina State University, Raleigh, NC 27695, USA; ^2^ Genetics Program, North Carolina State University, Raleigh, NC 27695, USA; ^3^ Toxicology Program, North Carolina State University, Raleigh, NC 27695, USA; ^4^ Center for Human Health and the Environment, North Carolina State University, Raleigh, NC 27695, USA

**Keywords:** Cadmium, Developmental exposure, Exposure models, Fetal growth restriction, Disease programming, Organ systems

## Abstract

Cadmium (Cd) is a naturally occurring toxic heavy metal found ubiquitously throughout the environment. Anthropogenic activities since the onset of industrialization have led to widespread environmental contamination that has substantially increased human exposure and associated health risks. As one of the top ten chemicals of major public health concern of the World Health Organization, Cd poses significant risks to human health, particularly when exposure occurs during the critical stages of development. Cd accumulates in the placenta and can be detected in cord blood and fetal and neonatal tissues, so it is crucial to understand the consequences of early-life Cd exposure and the underlying molecular mechanisms. In this Review, we provide an overview of the models currently used to study developmental Cd exposure and integrate the findings from epidemiological, animal and *in vitro* studies. We explore the impacts and mechanisms of early-life Cd exposure on the placenta, growth and development, and organ systems, identifying common themes across diverse model systems. Finally, we pinpoint knowledge gaps and propose key research priorities that will advance our understanding and inform mitigation strategies for reducing the developmental risks of Cd exposure.

## Teaser

**Summary:** This Review provides an account of the current scientific knowledge regarding the effects of cadmium exposure during early life on development and health outcomes in major organ systems.

## Introduction

Cadmium (Cd) is a heavy metal and non-essential trace element that is toxic to nearly all species (Andresen and Küpper, 2013; Dutta et al., 2020; Jacobson et al., 1981; Thévenod and Lee, 2013; Trevors et al., 1986; Xu and Morel, 2013). Cd naturally occurs in the Earth's crust at low levels [0.1-0.5 parts per million (ppm)] and is released into the environment through both natural processes (volcanic eruptions, fires, etc.) and human activities (mining, manufacturing, etc.) [Agency for Toxic Substances and Disease Registry (ATSDR), 2012; Cullen and Maldonado, 2013]. Over the past century, there has been a surge in industrial Cd consumption, the majority of which is driven by the manufacturing of nickel-Cd rechargeable batteries (Cullen and Maldonado, 2013).

**Fig. 1.**
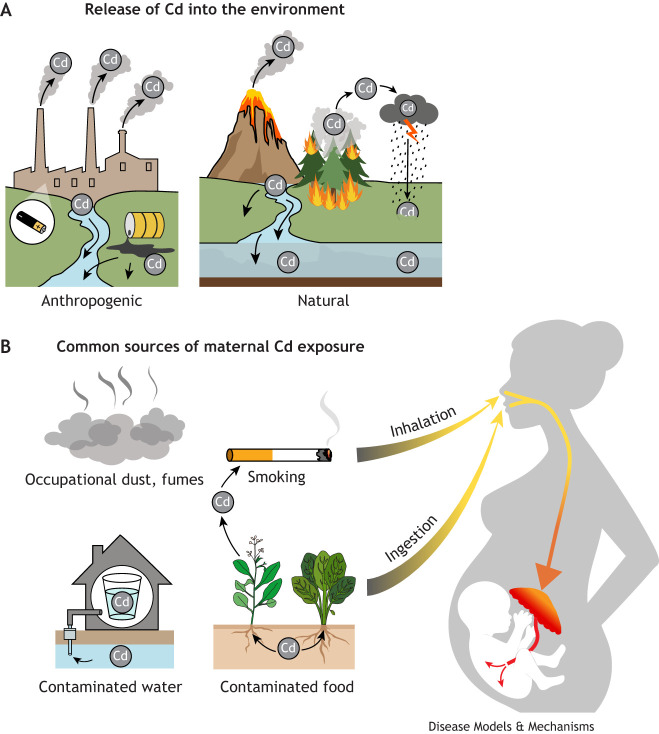
**An overview of the common sources and routes of cadmium (Cd) exposure.** (A) Cd is released into the environment through human activity, including industrial processes required for the production of nickel-Cd batteries. Cd can enter air and water from factory exhaust and accidental spills. Natural processes, including volcanic eruptions and wildfires, also emit Cd, which contaminates air through direct aerosolization and enters the water cycle. (B) Common sources of maternal Cd exposure include dust and fumes in occupational settings, and tobacco products, which are absorbed through the inhalation route. Cd is also ingested from contaminated drinking water, typically from unregulated private wells, and foods like leafy greens, which accumulate Cd. Both inhaled and ingested Cd can be absorbed and affect placental and fetal development.

Humans are predominantly exposed to Cd through two routes: inhalation and ingestion (Fig. 1) (ATSDR, 2012). Around 6% of ingested Cd is absorbed through the intestines, while 10-50% of inhaled Cd is absorbed through the lungs (Genchi et al., 2020; Järup, 2002; Mikolić et al., 2015). Tobacco plants efficiently uptake Cd from the soil, and, consequently, the Cd body burden of smokers is approximately double that of non-smokers (ATSDR, 2012; Cullen and Maldonado, 2013; Satarug et al., 2003). Cd can also be inhaled through contaminated dust or fume particles present in occupational settings (Cullen and Maldonado, 2013). In non-smokers, ingestion is the primary source of exposure and occurs predominantly through Cd-contaminated food (Kim et al., 2019) and water from unregulated private wells (Idrees et al., 2018; Kubier et al., 2019; Sanders et al., 2014).

Regardless of the exposure route, Cd is rapidly removed from the bloodstream and stored in tissues, with a biological half-life of ∼26 years (Genchi et al., 2020; Waalkes, 2003). The liver and kidneys are the main targets for Cd accumulation, with the latter storing upwards of 50% of the total body burden (Genchi et al., 2020). Pregnancy is a particularly vulnerable state because the programmed increase in essential nutritive metal uptake to support fetal demands leads to a concurrent increase in absorption of Cd (Delaney et al., 2020; Olsson et al., 2002; Satarug et al., 2010; Vahter et al., 2002). Interestingly, cord blood levels of Cd are much lower than those seen in maternal blood or the placenta, suggesting that there is limited Cd transport into fetal tissues (Dong et al., 2023).

In this Review, we summarize the utility of epidemiological studies, animal models and *in vitro* approaches (Box 1) and discuss findings that assess the effects of developmental Cd exposure. We present the current evidence linking Cd exposure to gestational and birth outcomes, including placental insufficiency, fetal growth restriction (FGR) and congenital anomalies, followed by an exploration of dysregulation and dysfunction in major organs and organ systems: the cardiovascular system, nervous system, reproductive system, liver and kidneys. Finally, we address the gaps in our current knowledge and propose areas that warrant further study.

Box 1. Approaches used for investigating the effects of developmental cadmium (Cd) exposure **Perinatal epidemiological studies** aim to understand the maternal, prenatal and postnatal health outcomes of events that occur during pregnancy and are often used to investigate the health effects of developmental Cd exposure (Buck Louis and Platt, 2011; Dunne et al., 2021; Giannakou, 2021). Given that these studies are observational, they typically use a cohort design in which pregnant women, child-mother pairs and/or children are recruited based on known or possible Cd exposure. Alternatively, they employ a case-control design in which recruitment is based on the occurrence of a specific health impairment (Chandravanshi et al., 2021; Song and Chung, 2010). Cd can be non-invasively quantified in maternal or fetal biological specimens (e.g. blood, urine, hair, cord blood, placenta, breast milk) to determine whether an observed phenotype can be attributed to Cd exposure (Vilahur et al., 2015).

**Experimental animal studies** enable specific hypothesis testing of the mechanisms underlying developmental Cd toxicity (Parasuraman, 2011). Rodents are most commonly used in developmental studies to model human exposure as they share similar anatomical structures (e.g. placenta), physiological processes and molecular pathways during development (Alberts et al., 2002; Carter, 2020). In developmental Cd studies, female rodents are administered Cd before and/or during pregnancy which permits investigation of the combined effects of Cd exposure on maternal physiology and direct transfer of Cd to the fetus (King-Herbert and Vasbinder, 2020; Leroy and Allais, 2013; Marsden and Leroy, 2013; Parasuraman, 2011). Fish, chickens and frogs are also used in studies to determine the direct effects of Cd exposure on developing offspring, as their *ex utero* development and semi-transparent embryos allow for easier exposure, visualization and manipulation (Liu et al., 2016b; Stark and Ross, 2019; Veldman and Lin, 2008).

***In vitro* models** have been used in toxicology research for decades as they are cost effective, use few to no live animals, and require less time and physical space. Common *in vitro* models include cultured cells, micromass, organs, tissues and whole embryos (Winn, 2019; Lee et al., 2012; Pamies et al., 2011). The simplified nature of *in vitro* models allows direct investigation of the effects of Cd and facilitates precise control of physical, chemical and genetic parameters (Allen et al., 2008; Brannen et al., 2016; Winn, 2019; Il'yasova et al., 2015; Lee et al., 2012; Pamies et al., 2011). However, mimicking the gestational environment in placental animals is challenging when using *in vitro* methods; therefore, *in vitro* models can complement animal models to further explore the mechanisms driving the response to developmental Cd exposure.

## Cd transport, accumulation and effects on the placenta

In mammals, the placenta acts as a semi-permeable barrier to the maternal-fetal exchange of Cd and is where most Cd accumulates (Barrett et al., 2023). Only a small amount of Cd enters fetal circulation compared to other metals like lead (Pb) and mercury (Hg) (Kantola et al., 2000; Plöckinger et al., 1993; Sakamoto et al., 2013). The amount of Cd that crosses the placental barrier can vary based on the dose and route of exposure, gestational age, fetal sex, maternal health, diet, lifestyle and other factors (Ahokas and Dilts, 1979; Jackson et al., 2022; Sonawane et al., 1975; Dong et al., 2023). Notably, early rodent studies found that fetuses could tolerate direct Cd injections at concentrations that were embryonic lethal when administered to the mother (Levin et al., 1981), indicating that the impacts of maternal Cd exposure are more likely due to impaired placental function and/or poor gestational environment rather than direct effects of Cd within fetal tissues.

**Fig. 2.**
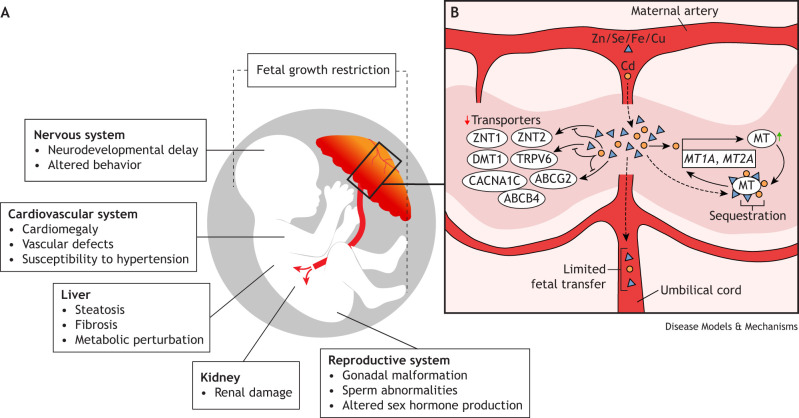
**A summary of the impacts of developmental cadmium (Cd) exposure on the placenta and multiple organ systems.** (A) Maternal Cd exposure is linked to fetal growth restriction and affects multiple organ systems, including the nervous, cardiovascular and reproductive systems, as well as the liver and kidneys. (B) Cd (orange circles) disrupts homeostasis of essential trace metals, including zinc (Zn), selenium (Se), iron (Fe) and copper (Cu) (blue triangles). The presence of Cd in the placenta induces upregulation of metallothionein (MT), which non-specifically sequesters metals. Additionally, developmental Cd exposure has been shown to interfere with specific transporters, including zinc transporter (ZNT)1 (also known as SLC30A1), ZNT2 (also known as SLC30A2), divalent metal transporter 1 (DMT1; also known as SLC11A2), transient receptor potential cation channel subfamily V member 6 (TRPV6), voltage-dependent L-type calcium channel subunit alpha-1C (CACNA1C), ATP-binding cassette super-family G member 2 (ABCG2) and ATP-binding cassette, subfamily B, member 4 (ABCB4). Together, these perturbations limit the transfer of both Cd and essential trace metals to the developing fetus.

Cd is sequestered in the placenta owing to a high affinity for metal-binding metallothionein (MT) (see Glossary, Box 2) proteins (Coyle et al., 2002) (Fig. 2). Placental Cd-MT accumulation triggers a positive feedback loop, whereby MT synthesis is upregulated. Because of the non-specific affinity of MT for metal binding, zinc (Zn), copper (Cu), iron (Fe) and selenium (Se) also become sequestered in the placenta, reducing transfer of these essential nutrients to the fetus (Coyle et al., 2002; Waalkes and Goering, 1990). Higher placental Cd levels are associated with reduced Zn transport to the fetus, regulated by placental zinc transporter (*Znt*)*1* (also known as *Slc30a1*) and *Znt2* (also known as *Slc30a2*) (Wang et al., 2016b). Cd may also interfere with placental transporters for Fe [e.g. divalent metal transporter 1 (DMT1; also known as SLC11A2)] (Leazer et al., 2002; Somsuan et al., 2019) and calcium (Ca) [e.g. transient receptor potential cation channel subfamily V member 6 (TRPV6) and voltage-dependent L-type calcium channel subunit alpha-1C (CACNA1C)] (Phuapittayalert et al., 2016). In addition, Cd exposure has been shown to reduce the abundance of placental transporters ATP-binding cassette super-family G member 2 (ABCG2) and ATP-binding cassette, subfamily B, member 4 (ABCB4), which are responsible for translocating heavy metals from the placental cell membranes back into maternal circulation (Liu et al., 2016a). Recent *in vitro*, *in vivo* and epidemiological studies have further bolstered the role of placental ABCG2 in regulating maternal Cd toxicity; reduced efflux efficiency conferred by the Q141K variant was found to be less protective against the Cd-induced activation of stress genes and cytotoxicity *in vitro* (Wen et al., 2021) and to increase the strength of the inverse relationship between placental Cd concentration and placental weight in newborns (Barrett et al., 2023). Cd-exposed *Abcg2* knockout mice had significantly higher renal Cd accumulation than wild-type mice owing to reduced urinary excretion (Wen et al., 2021).

Box 2. Glossary **11β-hydroxysteroid dehydrogenase 2 (11β-HSD2):** an enzyme responsible for limiting the transfer of the glucocorticoid cortisol from maternal circulation to the fetus by catalyzing the inactivating conversion of cortisol to cortisone.

**AKT signaling:** a signaling pathway that functions as a key regulator of cell growth, survival and metabolism.

**Cardiomegaly:** a condition characterized by enlargement of the heart, usually symptomatic of an underlying pathology.

**Ectrodactyly:** a rare birth defect that causes one or more fingers or toes to be missing or malformed.

**Glomerulus:** a cluster of capillaries in the kidneys responsible for filtering cells and proteins from the blood to create a glomerular filtrate, which ultimately becomes excreted as waste in the form of urine.

**Gonadotropin-releasing hormone:** a hormone released from the hypothalamus that triggers the production and secretion of luteinizing hormone and follicle-stimulating hormone from the pituitary gland. These produced hormones are important for regulating sex-specific hormone production in the gonads.

**Granulosa cells:** a follicular cell type located in the ovaries that clusters around the developing egg and provides estrogen and progesterone at key points in the menstrual cycle.

**Implantation:** the first stage of gestation when a newly fertilized egg attaches to the uterine lining, establishing a pregnancy.

**Intraperitoneal:** within the membrane (peritoneum) that lines the abdominal cavity and covers the abdominal organs.

**Leptin:** a hormone predominantly secreted by adipose tissue that regulates energy balance through influencing appetite, feeding behaviors and satiety.

**Leydig cells:** an interstitial cell type located in the testes, responsible for providing testosterone and other androgens to support sperm production, sexual maturation and development of secondary sex characteristics.

**Luteinizing hormone:** a hormone that stimulates ovulation in females and the development of secondary sexual characteristics in men.

**Metabolic dysfunction-associated steatotic liver disease (MASLD):** a chronic and progressive liver condition that occurs when fat accumulates in the liver. It was previously known as nonalcoholic fatty liver disease (NAFLD).

**Metallohormone:** a description given to metals that appear to display steroid hormone-like activity, despite otherwise having no known biological function.

**Metallothionein:** a family of proteins that bind metals, including metals that are essential (e.g. zinc) and toxic (e.g. cadmium). Metallothionein protects against oxidative stress and metal toxicity.

**Orofacial cleft:** a birth defect that causes tissues of the lip and mouth to not form properly.

**Placenta previa:** a condition whereby the placenta attaches to the lower part of the uterus and partially or completely covers the cervix, often leading to preterm birth and maternal hemorrhage.

**Placentation:** the location and process of placenta formation following implantation of the embryo in the uterus, allowing for exchange of nutrients and oxygen from maternal to fetal circulation.

**Preeclampsia:** a serious high-blood pressure condition that can occur during pregnancy or after giving birth.

**Preneoplastic lesions:** abnormal cells that have a higher risk of developing into non-cancerous or cancerous tumors.

**Proximal tubule cells:** a major epithelial cell type in the kidneys that make up the proximal tubules, a substructure of the nephron responsible for reabsorbing fluid that has been filtered through the glomerulus back into circulation.

**Sertoli cells:** a structural cell type located in the seminiferous tubules of the testes, characterized by a columnar shape and very large nuclei, responsible for supporting sperm production.

**Striatal:** related to the striatum, a coordinated group of structures in the forebrain with critical roles in cognition, motor function and learning.

**Teratogenic:** a substance or exposure that has the potential to cause abnormal development in an embryo or fetus.

**Transferrin:** a family of glycoproteins involved in the transfer and homeostasis of iron.

**Ubiquitination:** the process by which ubiquitin proteins are attached to target proteins, marking them for degradation. This process is important for maintaining proper transcriptional regulation, cell surface signaling and regeneration.

**WNT/β-catenin pathway:** a signaling pathway that plays critical roles in embryonic development and adult tissue homeostasis.

## Placental Cd exposure and FGR: pathology and mechanisms

Early-life Cd exposure has been consistently linked with FGR and low birth weight (LBW) in epidemiological studies (Table S1) (Röllin et al., 2015; Tian et al., 2009; Vidal et al., 2015; Zhang et al., 2004) and animal models (Table S2) (Castillo et al., 2012; Enli et al., 2010; Hudson et al., 2021; Ronco et al., 2011; Thompson and Bannigan, 2001; Zhang et al., 2012, 2015), in both placental and non-placental species. FGR is typically concurrent with placental insufficiency, wherein the placenta does not adequately support the growing fetus (Nardozza et al., 2017) and increases the risk of premature birth, sudden infant death syndrome, metabolic disorders, impaired neurodevelopment and heart disease (Sabra et al., 2017), highlighting the importance of mitigating exposure to Cd and other potential FGR risk factors during pregnancy (Fig. 2). Regardless of dose, maternal intraperitoneal (Box 2) injection during gestation more consistently leads to FGR and LBW (Fernandez et al., 2003; Ji et al., 2011; Zhang et al., 2016). However, growth restriction is generally only observed following maternal ingestion of water containing ≥30 ppm Cd (Ronco et al., 2009, 2011; Zhao et al., 2018; Hudson et al., 2019), suggesting a dose-dependent threshold to induce FGR and LBW. Female offspring appear to be more susceptible to Cd-impaired fetal growth, particularly in studies with large cohorts or those conducted in areas with notable Cd contamination (Kippler et al., 2012; Cheng et al., 2017; Taylor et al., 2016; Zhang et al., 2018a), indicating that there may be subtle sex-specific differences that become more pronounced at higher doses. Co-administration of Cu or Zn can prevent FGR/LBW (Enli et al., 2010; Fernandez et al., 2003), and, considering that Cd is able to bind essential metal transporters, it is likely that FGR and LBW are consequences of altered essential trace metal homeostasis.

Understanding the pathological impacts of Cd exposure on FGR requires a comprehensive examination of its impact on the placenta. Numerous studies have shown impacts of maternal Cd exposure on placental structure and function, including implantation (Box 2), nutrient transport, hormone signaling, oxidative stress, and genomic and epigenomic effects, which are discussed in detail below and summarized in Tables S3-S5.

### Impaired placentation and implantation

Cd reportedly impacts the earliest stages of fetal development by affecting the process and site of placentation (Box 2) and implantation during early development. In support of this, women with placenta previa (Box 2) had higher blood Cd levels (Tsuji et al., 2019). Furthermore, activities of the implantation enzymes cathepsin-D and alkaline phosphatase were downregulated or upregulated, respectively, by maternal Cd exposure in rats (Nampoothiri and Gupta, 2008). Cd accumulates in the labyrinth zone of the mature placenta of rodents, which is important for maternal-fetal nutrient and gas exchange (Yamagishi et al., 2016). Trophoblasts in the placental labyrinth zone display reduced proliferation, increased apoptosis and a muted response to differentiation induction, leading to placental insufficiency and FGR (Erboga and Kanter, 2016; Lee et al., 2009; Wang et al., 2012; Wier et al., 1990; Yamagishi et al., 2016; Zhou et al., 2016).

### Fetomaternal endocrine disruption

Cd exposure during pregnancy changes the abundance of maternal and fetal hormones, including glucocorticoids and steroid hormones that drive differentiation in favor of proliferation, leading some to assign Cd the classification of metallohormone (Box 2) (Wyrwoll and Holmes, 2012). Fetal exposure to elevated glucocorticoids is associated with LBW, and Cd was found in rodents to increase glucocorticoid concentrations in the fetus, placenta and maternal plasma (Ronco et al., 2009). Cd may lead to increased glucocorticoids through reduced expression, protein abundance and enzymatic activity of 11β-hydroxysteroid dehydrogenase 2 (11β-HSD2; also known as HSD11B2; Box 2), a negative regulator of maternal-fetal glucocorticoid transfer, as this has been observed both *in vitro* in human placental trophoblast cells and *in vivo* in rats (Wang et al., 2014; Yang et al., 2006). The role of 11β-HSD2 in Cd-induced FGR might depend on gestational timing, route and duration of maternal exposure, as well as the functionality of other mechanisms that mitigate Cd toxicity in the placenta. For example, one study found that placental 11β-HSD2 was seemingly unaffected by midgestational maternal Cd exposure in both MT-null and wild-type mice despite FGR occurring in the highest-dosed MT-null pups (Selvaratnam et al., 2013). An explanation offered for the lack of observed effects on 11β-HSD2 during Cd-induced FGR was that the effects are more pronounced in late gestation after 11β-HSD2 expression has peaked. Other Cd-induced impairments to placental glucocorticoid signaling may occur through altered glucocorticoid receptor expression, as one study identified Cd-associated changes to placental DNA methylation of nuclear receptor subfamily 3 group C member 1 (*NR3C1*), a glucocorticoid receptor gene (Appleton et al., 2017). Cd exposure is also associated with preeclampsia (Box 2), which may be linked to abnormal glucocorticoid homeostasis in the placenta, although a causative mechanism has not been established (Brooks et al., 2016; Wang et al., 2014, 2018; Zhang et al., 2016).

Cd exposure disrupts the synthesis and regulation of other key placental hormones that are critical for fetal development and maternal health, such as placental lactogens (Lee et al., 2009) and leptin (Box 2). Synthesis of progesterone, a hormone required for pregnancy maintenance and fetal development, is also disrupted by Cd exposure *in vitro* and *in vivo* (Jolibois et al., 1999a; Kawai et al., 2002). Accordingly, placentae from smokers have higher levels of Cd and lower levels of progesterone (Piasek et al., 2002). The underlying mechanisms leading to impaired placental progesterone synthesis may involve a Cd-induced reduction in placental cholesterol levels, altered expression of the low-density lipoprotein receptor or reduction of steroidogenic enzymes (Jolibois et al., 1999b; Nampoothiri and Gupta, 2008). Progesterone supplementation in pregnant rats can alleviate Cd-induced placental abnormalities and preeclampsia-related symptoms (Zhang et al., 2018b). Placental estrogen synthesis is similarly impaired and can also be ameliorated by supplementation with synthetic estrogen (Liu et al., 2022).

### Oxidative stress

Increased placental oxidative stress (OS) is often seen in the pathology of pregnancy-related disorders like preeclampsia and FGR. Elevated OS impairs trophoblast invasion and differentiation, triggers increased apoptosis of trophoblasts and induces placental endoplasmic reticulum (ER) stress (Wang et al., 2012; Wu et al., 2015). Gestational exposure to Cd has been shown to exacerbate placental biomarkers of OS, including free radicals, oxidative DNA damage, and DNA damage repair proteins apurinic/apyrimidinic endonuclease 1 (APE1; also known as APEX1) and p53 (also known as TP53), along with a reduction in total antioxidant capacity (Zhang et al., 2016). Additionally, the key antioxidant glutathione is reduced in Cd-exposed rodent placentae (Enli et al., 2010; Wang et al., 2012). Disruption of placental glutathione metabolism may contribute to preeclampsia (Jin et al., 2017). *In vivo* work suggests that supplementation with N-acetylcysteine, a glutathione precursor, can rescue the negative effects of Cd on placental development, gene expression, ER stress and FGR (Guo et al., 2018).

Cd-induced reactive oxygen species (ROS) further disrupt placental development by activating hypoxia-inducible factor 1 (HIF-1; also known as HIF1A), a key regulator of oxygen homeostasis, and associated HIF-1 targets, and by altering expression of transforming growth factor beta (TGF-β) pathway members that are required for trophoblast differentiation and invasion (Adebambo et al., 2018). In addition, Cd exposure activates the OS-sensitive transcription factor nuclear factor kappa-light-chain-enhancer of activated B cells (NF-κB), which plays a central role in placental inflammatory responses (Ronco et al., 2011). Mechanistically, Cd promotes placental inflammation at least in part through activating the AKT serine/threonine kinase (AKT) signaling (Box 2) pathway both *in vivo* and *in vitro*; inhibition of this pathway has been shown to alleviate Cd-induced upregulation of pro-inflammatory cytokines (Hu et al., 2018). Antioxidant supplementation during Cd exposure has been shown to alleviate Cd-induced OS and related phenotypes in both the placenta and fetus, supporting the notion that increased OS contributes to the pathophysiology of maternal Cd exposure and providing promising support for antioxidant therapies (Adebambo et al., 2018; Enli et al., 2010; Guo et al., 2018; Wang et al., 2012).

### Disrupted carbohydrate metabolism

Glycogen, the storage form of glucose, is abundant in the placenta during early pregnancy, although its exact role remains debated. However, elevated placental glycogen levels are often observed in poorly functioning placentae associated with FGR and preeclampsia (Akison et al., 2017; Arkwright et al., 1993). Maternal Cd exposure has been associated with elevated placental glycogen levels during late rodent gestation when levels would typically decline (Roelfzema et al., 1987, 1988a,b, 1989; Yoruk et al., 2003). Impaired enzymatic activity of glycogen phosphorylase, which breaks down glycogen into glucose, might be expected to contribute to glycogen accumulation; however, one study in rats found increased placental glycogen phosphorylase activity following maternal Cd exposure, indicating higher glycogen turnover and disrupted downstream carbohydrate metabolism (Roelfzema et al., 1989). In support of this idea, glucose transporter 3 (*Glut3*; also known as *Slc2a3*) expression was found to be significantly reduced in the murine placenta owing to maternal Cd exposure (Xu et al., 2016). Additionally, Cd-induced maternal and fetal Fe deficiencies during gestation have been associated with hypoxia in newborn mouse tissues, and a hypoxic environment during development may impair aerobic respiration and shift energy production towards inefficient anaerobic pathways to meet the energy demands of developing fetal tissues (Hudson et al., 2019, 2021). In contrast, another study reported decreased placental glycogen levels after subcutaneous Cd administration throughout pregnancy (Nampoothiri and Gupta, 2008); however, this finding was accompanied by significant reductions in all other measured biomarkers, suggesting severe placental damage and a pre-apoptotic state.

### Altered regulation and stability of the genome and epigenome

Maternal Cd exposure also exerts effects on the placental genome and epigenome. Elevated placental Cd is associated with shortened telomere length, a hallmark of cancer and aging (Lin et al., 2013). Similarly, an *in vitro* model of embryonic exposure to cigarette smoke also demonstrated reduced telomere length (Huang et al., 2010). Cd has also been shown to damage DNA *in vitro* through OS (Nagaraju et al., 2022; Skipper et al., 2016). Female rats treated with Cd during pregnancy exhibited a significant reduction in placental DNA and RNA content (Lee et al., 2009; Nampoothiri and Gupta, 2008; Zhang et al., 2016), consistent with Cd-induced placental DNA damage.

Developmental Cd exposure is also associated with altered fetal DNA methylation in humans and animal models (Kippler et al., 2013; Sanders et al., 2014; Zhu et al., 2011). In humans, higher maternal Cd exposure is associated with altered fetal DNA methylation at genomic loci implicated in body weight and at imprinted genes that contribute to fetal growth (Cowley et al., 2018). In fact, altered DNA methylation at specific loci has been linked to maternal Cd burden and birth weight in the same population (Kippler et al., 2013). In the placenta, Cd-associated differentially methylated regions (DMRs) have been found in or near genes with roles in inflammatory signaling, cell growth, birth metrics, hormone receptors and fetal growth (Appleton et al., 2017; Everson et al., 2016, 2018). Some placental DMRs are sex specific; in female offspring, Cd-associated DMRs were identified in promoters of genes associated with cell damage response, whereas in males the DMRs were associated with genes involved in cell differentiation, angiogenesis and organ development (Mohanty et al., 2015). However, a genome-wide analysis of DNA methylation in the mouse placenta did not identify any DMRs associated with Cd exposure, suggesting that Cd exerts species-specific effects on the placental epigenome, possibly reflecting differences between murine and human placental structure (Simmers et al., 2023). The aforementioned approach does not rule out Cd-induced differential methylation in mice; additional studies are needed to determine whether this was inherent to the study design or whether there are truly species-specific differences in how Cd affects the placental epigenome. Differences between murine and human placental structure and gene expression patterns have been documented, so novel techniques like three-dimensional placenta organoids and embryoid bodies provide promising research platforms to accurately study the impact of Cd exposure on the placenta (Hemberger et al., 2020).

## Effects of developmental Cd exposure on fetal tissues and organ systems

The preceding sections highlight the critical role of the placenta in the early pathogenesis of adverse health outcomes owing to maternal Cd exposure; the following sections will explore the effects on non-placental tissues and organ systems.

### Congenital anomalies

Evidence from experimental animal models suggests that Cd is teratogenic (Box 2); however, this is not well substantiated in humans (Calle et al., 2015). One recent study in humans connected placental Cd levels to newborn orofacial clefts (Box 2), but the simultaneous presence of Pb and Hg made it unclear whether Cd alone was responsible (Pi et al., 2018). In rodent studies, teratogenic effects depend on the dose, route and gestational timing of Cd administration and genetic background (Table S6). When pregnant female rodents are exposed orally to Cd, there do not appear to be teratogenic effects (Barański et al., 1982; Saxena et al., 1986; Wardell et al., 1982). However, when Cd is administered to pregnant female rodents through intravenous or intraperitoneal injection, congenital anomalies are nearly always observed (Chen et al., 2008; Elsaid et al., 2010; Fernandez et al., 2003; Holt and Webb, 1987; Murata et al., 1993; Padmanabhan, 1987; Samarawickrama and Webb, 1979; Webb et al., 1988; Webster and Messerle, 1980). Teratogenic effects of Cd have also been reported in zebrafish and chick embryos, likely owing to the direct route of Cd exposure in an aqueous medium (Bosch et al., 2023; Chouchene et al., 2022; Di Paola et al., 2022; Hen Chow et al., 2009; Manigandan et al., 2015; Thompson and Bannigan, 2008; Zhang et al., 2015). Cd-induced defects in non-mammalian models appear to depend on the dose and timing of exposure; low concentrations cause no observable abnormalities (Di Paola et al., 2022), whereas higher doses consistently induce malformations (Chow et al., 2008; Zhang et al., 2015). Cd telluride (CdTe) dots, nanoparticles that have many applications in science and technology, are also linked to spinal defects, although the role of Cd remains unclear because the severity of the effect is dependent on the selected functionalization compound (Bosch et al., 2023).

Multiple pathways underlie the teratogenic effects of Cd. In mouse embryos, maternal intraperitoneal injection of Cd during neurulation altered the expression of genes involved in metabolism, transcription, translation, mitochondrial organization, cell cycle and stress response; genes that were significantly differentially expressed depended on the Cd dose and post-injection latency (Robinson et al., 2011). In rodents, Cd-induced forelimb ectrodactyly (Box 2) may be caused by disruption to fibroblast growth factor (FGF) and sonic hedgehog (SHH) signaling pathways (Elsaid et al., 2007; Schreiner et al., 2009). Disruption of SHH-directed anterior-posterior patterning of vertebrate limbs could explain why increasing doses of Cd lead to increasingly anterior forelimb ectrodactyly (Scott et al., 2005). SHH signaling may be further inhibited by a Cd-induced perturbation of Zn, a required structural component of the SHH protein (Schreiner et al., 2009). Abnormal levels of retinoic acid during development is a well-established cause of congenital malformation, and Cd has been shown to alter its signaling in cultured cells and in mice (Cui and Freedman, 2009; Hudson et al., 2021). Cd-induced developmental defects in zebrafish parallel those seen with retinoic acid depletion, and retinoic acid supplementation can rescue some of the Cd-induced phenotypes (Zhang et al., 2015).

### Cardiovascular system

Developmental exposure to Cd can impair cardiovascular development and influence susceptibility to cardiovascular disease (CVD) (Table S7), although findings vary between animal models and human studies. In mice, maternal Cd exposure results in cardiomegaly (Box 2) and reduced myocyte numbers, alongside altered expression of genes linked to WNT/β-catenin signaling and hypertension (Hudson et al., 2019; Omidi et al., 2019). In contrast, human cardiac organoids derived from embryonic stem cells display reduced differentiation and suppression of the WNT/β-catenin pathway (Box 2) (Wu et al., 2022).

Evidence from rodent models supports the long-term cardiovascular consequences of developmental Cd exposure. Rodents born to Cd-exposed mothers have altered heart morphology, vascular reactivity and susceptibility to hypertension in adulthood (Hudson et al., 2019; Ronco et al., 2011). Female mice exposed to Cd from conception to adulthood, in combination with a high-fat diet after weaning, develop cardiac hypertrophy and fibrosis, although the contribution of prenatal Cd exposure to the extent of this phenotype is unclear (Liang et al., 2019). Paradoxically, one study suggested that maternal Cd exposure may aid in resistance to ischemic injury during myocardial infarction in adult rats (Zepeda et al., 2012).

Non-placental experimental models further highlight the detrimental effects of Cd on cardiovascular development (Table S7). Cd-exposed zebrafish embryos display abnormal vasculature, reduced complexity of craniofacial vasculature and reduced heart rates (Cheng et al., 2001; Zhang et al., 2012). Embryonic Japanese medaka fish exposed to Cd-contaminated sediment developed elevated heart rates during development and cardiovascular abnormalities at hatching (Barjhoux et al., 2012). In frog tadpoles, exposure to a low dose of Cd considered in Brazil to be environmentally safe depressed cardiac functions (Dal-Medico et al., 2014).

Although there is strong experimental evidence that Cd negatively impacts cardiovascular development in animals, there are limited findings from epidemiological studies. One study found no association between maternal urinary Cd during pregnancy and blood pressure in 4.5-year-olds (Hawkesworth et al., 2013); another found no association between blood Cd in 2-year-olds and blood pressure throughout childhood (Cao et al., 2009). In humans, Cd may have additive or synergistic effects with other heavy metals that affect cardiovascular development and function. One study reported that elevated maternal blood Cd, in combination with Pb, was associated with increased offspring risk for congenital heart defects, although no significant association was observed for Cd alone (Ou et al., 2017). Another study found that high maternal Cd exposure, as measured in maternal hair samples, was associated with an increased risk of congenital heart defects that was more pronounced when combined with arsenic exposure (Jin et al., 2016). It is also possible that the cardiotoxic effects of developmental Cd exposure are unique to rodent models at high doses that are rarely encountered in human populations, although additional epidemiological studies are required to support this assertion.

Malnutrition during development is associated with increased CVD risk (Wadhwa et al., 2009), and changes to essential trace element homeostasis during prenatal development may be a contributing factor to Cd-associated CVD (Hudson et al., 2019; Mikolić et al., 2015). For example, fetal Fe deficiency induced by maternal Cd exposure may impair fetal oxygen homeostasis (Hudson et al., 2019, 2021) and indirectly lead to OS, a proposed direct mechanism of Cd-induced cardiotoxicity (Almenara et al., 2013). Exposure to Cd during early postnatal life may further impair Fe metabolism in cardiovascular tissues. In postnatally developing rats, Cd increased cardiac transferrin (Box 2) uptake independent of dietary Fe supply (Crowe and Morgan, 1997). Early postnatal Cd exposure may also affect Zn and Cu homeostasis in the heart; Cd-exposed chick embryos displayed delayed vasculogenesis and lacked clearly defined vessels, a phenotype that could explain Cd-associated malnutrition in the embryo (Gheorghescu and Thompson, 2016).

Epigenetic alterations offer another possible mechanism. At the epigenetic level, Cd-associated DMRs were identified in newborn human cord blood at loci involved in cardiometabolic functions, indicating a potential mechanism through which maternal Cd exposure may program long-term susceptibility to CVD (Cowley et al., 2018). However, mechanistic knowledge is lacking, and additional work is needed to identify causal mechanisms linking early-life Cd exposure with impaired cardiovascular development and disease susceptibility.

### Nervous system

Extensive epidemiological research links developmental Cd exposure to neurodevelopmental delays and disorders (Table S8). Higher maternal Cd levels correlate with a greater risk of emotional problems and diminished performance in cognition, perception, quantitation, locomotion, intelligence and social development tests (Bonithon-Kopp et al., 1986; Jeong et al., 2015; Kippler et al., 2012, 2016; Sioen et al., 2013; Tian et al., 2009; Wang et al., 2016a). Sex-specific differences have been reported, although it is unclear whether males or females are more sensitive (Ciesielski et al., 2012; Kippler et al., 2012; Sioen et al., 2013). Conversely, others report no significant relationship between maternal, newborn or juvenile Cd levels and impaired neurodevelopment or behavior, possibly owing to differences in behavioral testing and Cd sampling methodologies (de Assis Araujo et al., 2022; Freire et al., 2018; Garí et al., 2022).

Numerous animal studies have described the neurodevelopmental effects of early-life Cd exposure (Table S9). Maternal Cd has been linked with altered brain sizes when measured at birth or during postnatal development (Antonio et al., 1998; Gulati et al., 1986; Gupta et al., 1991; Hudson et al., 2021; Mikolić et al., 2016; Mimouna et al., 2018; Xu et al., 1993; Zhang et al., 2009), as well as altered behaviors, e.g. activity levels, exploration, sensorimotor skills, aversion or avoidance, grooming, cognitive development, learning, memory, stress, anxiety, aggression and sensitivity to sound (Ali et al., 1986; Allam et al., 2016; Barański, 1986; Barański et al., 1983; Dési et al., 1998; Feng et al., 2019; Gulati et al., 1986; Halder et al., 2016, 2019; Hudson et al., 2021; Lehotzky et al., 1990; Mimouna et al., 2018; Minetti and Reale, 2006; Nagymajtényi et al., 1997; Newland et al., 1986; Petersson Grawé et al., 2004; Rai et al., 2010; Rastogi et al., 1977; Zhao et al., 2018). There is also evidence in rodents that paternal Cd exposure may affect offspring behavior and neurodevelopment (Zhao et al., 2015), although the underlying mechanisms are unclear.

Evidence for Cd-impaired neurodevelopment in animal studies is often highly spatiotemporally dependent. In one study, gestational exposure alone was sufficient to induce neuronal degeneration in the cortex and hippocampus in gestational day (GD)20 fetuses (Allam et al., 2016), while a separate cross-fostering study found that both gestational and lactational exposure was required to elicit morphological changes to the hippocampus (Zhao et al., 2018). Some studies suggest that exposure cessation at birth mitigates adverse effects compared to exposure continuing through lactation (Liapi et al., 2013; Mikolić et al., 2016; Yargiçoğlu et al., 1997; Zhao et al., 2018). Multiple rodent studies found that maternal Cd exposure damaged subpopulations of neuronal cells and altered the architecture of multiple regions of the brain (Allam et al., 2016; Bekheet, 2011; Ben Mimouna et al., 2018b; Feng et al., 2019; Zhao et al., 2018). Hydrocephaly has also been linked to late-gestation Cd injection in rat fetuses (White et al., 1990).

Early-life Cd exposure alters lipid metabolism in ways that increase brain OS (Gupta et al., 1995, 1996; Gutiérrez-Reyes et al., 1998; Xu et al., 1993; Zhang et al., 2009; Zhao et al., 2015) and are more prominent when exposure continues into the postnatal period (Agar et al., 2000; Gulati et al., 1986; Gupta and Shukla, 1996; Yargiçoğlu et al., 1997). Increased ROS and impaired antioxidant response linked to lipid peroxidation are implicated in brain injury, inflammation and cell death (Anthonymuthu et al., 2016) and could be one mechanism through which developmental Cd exposure exerts neurotoxic effects. Concordantly, supplementation of early-life Cd exposure with antioxidants has been neuroprotective (Allam et al., 2016; Bekheet, 2011; Ben Mimouna et al., 2018a,b; Halder et al., 2016, 2019; Mimouna et al., 2018; Mukherjee et al., 2010). Myelination is a unique form of lipid metabolism that is affected by Cd, although it is unclear whether exposure has a stimulatory or suppressive effect (Gupta and Shukla, 1996; Hudson et al., 2021).

Early-life exposure to Cd can affect essential trace metal homeostasis in the brain. Decreases in brain Zn levels in response to Cd may vary with Cd dose, route of exposure and, particularly, age of the individual, as some studies have noted a significant Cd-induced decrease in brain Zn in only a subset of the timepoints measured (Barański, 1986; Gupta et al., 1993; Mikolić et al., 2016). Meanwhile, other studies have observed no significant Cd-induced changes in brain Zn when measured at a single timepoint (Gulati et al., 1986; Sowa and Steibert, 1985). Regardless, supplemental Zn has a neuroprotective effect during developmental Cd exposure (Ben Mimouna et al., 2018a,b; Mimouna et al., 2018). Cu is also essential for brain development and function (Scheiber et al., 2014). Fewer studies have quantified brain Cu in response to developmental Cd exposure; but, in general, a decrease in brain Cu is observed (Barański, 1987; Gulati et al., 1986; Gupta and Shukla, 1996; Mukherjee et al., 2010; Sowa and Steibert, 1985; Thomas and Mushak, 1986). Fe is involved in numerous essential processes, including oxygen homeostasis, myelin production, neurotransmitter synthesis and cellular metabolism (Ward et al., 2014). Fe dysregulation is evident but complex, as animal models have found increases (Mukherjee et al., 2010), decreases (Mikolić et al., 2016) or no change (Gupta and Shukla, 1996; Sowa and Steibert, 1985) in brain Fe following Cd exposure. The brain is the body's most resource-demanding organ and requires Fe to support its high energy and metabolic needs (Ward et al., 2014). Maternal Cd exposure can lead to systemic Fe deficiency at birth (Hudson et al., 2019). In Fe deficiency, oxygen transport and cellular energy production are impaired, potentially leading to hypoxic environments within brain tissue. To compensate, the body may prioritize the brain's Fe, oxygen and nutrient supply through a phenomenon known as ‘brain-sparing’, which frequently coincides with FGR (Cohen et al., 2015).

In concert with perturbed metal homeostasis, developmental Cd exposure increases MT levels in the brain (Ben Mimouna et al., 2018a,b; Gutiérrez-Reyes et al., 1998; Mimouna et al., 2018). Artificially increasing MT levels during Cd exposure in early postnatal rats alleviated Cd-induced increases in striatal (Box 2) lipid peroxidation and dopamine release, possibly by increasing the available MT to bind Cd or Zn (Gutiérrez-Reyes et al., 1998). In support of a protective role for MT, MT-null mice were more sensitive to thyroid enzyme activation in the brain following early-life Cd exposure (Mori et al., 2006).

Rodent models have shown that early-life exposure to Cd can affect the levels of multiple neurotransmitters and hormones. The dopaminergic pathways – which are involved in motivation, reward-seeking behaviors and addiction (Volkow et al., 2017) – are perturbed by developmental Cd exposure, but the effects vary by area of the brain or exposure parameters (Allam et al., 2016; Antonio et al., 1998; Gutiérrez-Reyes et al., 1998; Rastogi et al., 1977). Serotonin signaling is implicated in depression and anxiety disorders, may influence reward and aversion behavior, and is affected by developmental Cd exposure in rodents (Hu, 2016; Szeitz and Bandiera, 2018). Rat pups orally dosed with Cd early in life had increased norepinephrine in multiple regions of the brain (Rastogi et al., 1977). Another study found that mice born to mothers who received Cd injections during gestation had low brain acetylcholine after weaning, which could be partially rescued by antioxidant-rich parsley juice supplementation (Allam et al., 2016). The activity of acetylcholinesterase, the enzyme that breaks down acetylcholine to terminate its neurotransmission, has also been found to be altered by maternal Cd exposure, although studies are inconsistent on whether the enzyme activity is increased or decreased (Liapi et al., 2013; Mukherjee et al., 2010; Stolakis et al., 2013).

Consistent with the classification of Cd as a metallohormone, developmental exposure has been shown to affect sex hormone signaling in the brain (Byrne et al., 2009). Mice born to mothers who ingested a moderate dose of Cd during pregnancy and through the first 10 days of postnatal life had reduced sex hormone receptors in their brains that were receptor and sex specific (Ishitobi et al., 2007). Synthesis of placenta-derived estrogen is impaired by Cd and linked to delays in neuronal development and escape behavior (Liu et al., 2022). Excess glucocorticoids can affect neurodevelopment and behaviors, and Cd increases fetal glucocorticoids and methylation of placental glucocorticoid receptor genes (Appleton et al., 2017). Perinatal Cd exposure has also been found to interfere with thyroid hormone metabolism in neonatal mice with MT deficiency (Mori et al., 2006).

Although less commonly used, non-mammalian and *in vitro* models provide valuable insight into Cd-induced dysneurogenesis (Chow et al., 2008; Gulisano et al., 2009; Hen Chow et al., 2009; Ohtani-Kaneko et al., 2008). Zebrafish embryos exposed to Cd exhibit reduced motor activity and hypersensitivity to auditory stimuli, and their brains appear smaller than those of zebrafish embryos without Cd exposure, with poorly defined regions (Chow et al., 2008). There is also evidence from zebrafish that implicates the WNT/β-catenin pathway and Ca signaling in impaired neurodevelopment (Green et al., 2023; Xu et al., 2022a,b). Proliferation of primary human fetal neuroblasts is altered in a dose-dependent manner, whereby a low dose of Cd increases growth, but a higher dose reduces proliferation and increases cell death (Gulisano et al., 2009). Meanwhile, primary fetal rat cortical cells exposed to Cd exhibit decreased dendritic and synaptic development, and decreased expression of proteins involved in neuronal development, with no change in cell proliferation (Ohtani-Kaneko et al., 2008). Thus, the effects of Cd on the nervous system are wide ranging and incompletely understood.

### Reproductive system

Knowledge of the impacts of developmental Cd exposure on the reproductive system is primarily derived from rodent studies (Table S10). Despite inefficient transfer across the placenta, Cd is detectable in testicular and ovarian tissues following developmental exposure (Bekheet, 2011; Chemek et al., 2016; Li et al., 2018a,b; Zhao et al., 2015). In general, Cd causes a reduction in gonad weight (Chemek et al., 2016; Ji et al., 2011; Liu et al., 2021; Luo et al., 2015; Samuel et al., 2011; Tam and Liu, 1985; Tian et al., 2018), although a handful of studies have shown little to no change (Li et al., 2018b; Luo et al., 2015; Pillai et al., 2012). The direct interaction of Cd with the gonads manifests in histological, morphological or structural changes in several cell types including germ cells, Sertoli cells (Box 2), Leydig cells (Box 2), granulosa cells (Box 2) and spermatogenic cells. One of the more notable effects in male offspring is altered diameter, structure and number of seminiferous tubules (Bekheet, 2011; Chemek et al., 2016; Corpas and Antonio, 1998; Huang et al., 2020; Liu et al., 2021). Production of sperm in the testes occurs in the seminiferous tubules, where disorganization or dysfunction can lead to reduced sperm counts, decreased motility or other sperm abnormalities (Banzato et al., 2012; Huang et al., 2020; Luo et al., 2015; Pillai et al., 2012; Zhang et al., 2019). Interestingly, maternal Zn supplementation during gestation and lactation has been shown to rescue testicular morphology and mitigate sperm abnormalities caused by maternal Cd exposure (Chemek et al., 2018). In female offspring, maternal Cd exposure alters ovarian follicles. Although it is unclear whether folliculogenesis is primarily promoted or impaired (Li et al., 2018b; Samuel et al., 2011), any dysregulation of folliculogenesis or normal follicular function can impact fertility (Chang and Cook-Andersen, 2013).

Developmental Cd exposure also affects reproductive hormone levels in blood serum and gonadal tissues. In males, exposure consistently reduces levels of reproductive hormones, including serum and testicular testosterone, serum gonadotropin-releasing hormone (Box 2), luteinizing hormone (Box 2) and progesterone (Chemek et al., 2016; Ji et al., 2011; Pillai et al., 2012). In females, findings are more variable; serum levels of testosterone, progesterone, estradiol and ovarian progesterone generally decrease following Cd exposure, although some studies report elevated serum estradiol and progesterone (Li et al., 2018b). Differences in serum and tissue hormone levels may be partially explained by changes in the expression of genes and enzymes involved in regulating steroidogenesis (Huang et al., 2020; Ji et al., 2011; Kariyazono et al., 2015; Liu et al., 2020, 2021; Tian et al., 2018), the process by which cholesterol is converted to steroid hormones in the gonads and other endocrine organs (Miller and Auchus, 2011). Notably, a consistent finding across studies is the co-downregulation of steroidogenic acute regulatory protein (StAR), the rate-limiting enzyme in the biosynthesis of steroid hormones, with testosterone and other steroids in both male and female gonads (Manna et al., 2009). One study reporting increased serum steroid hormones in females found a concurrent increase in ovarian StAR enzyme expression (Li et al., 2018b). These results suggest that Cd disrupts reproductive function by altering steroid hormone production.

Some studies showed that F1 females exposed to Cd during development had alterations in the day of vaginal opening: two studies found an earlier onset (Li et al., 2018b; Parodi et al., 2017), and two studies found delayed onset (Salvatori et al., 2004; Samuel et al., 2011), while others found no effect (Ishitobi and Watanabe, 2005; Luo et al., 2015). Despite these variations in the age of vaginal opening, fertility was unaffected. One study even found that exposed female F1 mice had larger litter sizes (Li et al., 2018b). A handful of studies have also shown that Cd exposure in F1 mice has detrimental effects on the reproductive systems of F2 and F3 generations (see Table S10) (Huang et al., 2020; Li et al., 2018b; Liu et al., 2020, 2021), highlighting the persistent intergenerational and transgenerational effects of Cd.

Although reproductive studies typically have focused on maternal exposures, paternal Cd exposure can also affect offspring reproductive health. A recent study in mice found that paternal exposure affected sex hormone synthesis and lipid metabolism in female offspring (Zhang et al., 2023). Whereas one rat study found that paternal Cd exposure negatively affected sperm quality in the male offspring (Zhao et al., 2015), a separate rat study found no changes in paternally exposed testes (Nan et al., 2020), suggesting potential species-, sex- or strain-specific sensitivities to paternal Cd exposure.

### Liver

Although animal studies have provided a wealth of evidence supporting a link between developmental Cd exposure and later-onset liver disease, including metabolic dysfunction-associated steatotic liver disease (MASLD; Box 2) (Table S11), few epidemiological studies have directly linked maternal Cd burden with offspring liver function. A handful of studies have examined the effect of maternal tobacco smoking on fetal liver and found molecular changes, but did not implicate Cd itself as a contributing factor (Drake et al., 2015; Filis et al., 2015; O'Shaughnessy et al., 2011).

Numerous rodent studies have shown that mice born to Cd-exposed mothers are deficient in hepatic Zn and Fe (Barański, 1986; Roelfzema et al., 1989; Hudson et al., 2019; Kuriwaki et al., 2005; Mikolić et al., 2016; Pillai and Gupta, 2005; Roelfzema et al., 1987; Sasser et al., 1985; Sorell and Graziano, 1990; Sowa and Steibert, 1985). Studies have also found perturbed homeostasis in other hepatic essential metal ion homeostasis (Hudson et al., 2019) that may be more pronounced in females (Jackson et al., 2022).

Maternal Cd exposure has been shown to disrupt metabolic pathways and functions in the developing liver, affecting insulin regulation (Jackson et al., 2020; Jacquet et al., 2019; Men et al., 2021; Mimouna et al., 2018), metabolism of sugars and glycogen (Roelfzema et al., 1987; Jackson et al., 2020; Pillai and Gupta, 2005; Stewart et al., 1984; Yi et al., 2021; Yoruk et al., 2003), hormone production and regulation (Castillo et al., 2012), and lipid peroxidation and deposition (Jackson et al., 2020; Xu et al., 1993). One study found that maternally exposed male mice had hyperglycemia and enhanced hepatic gluconeogenesis during puberty, the latter of which persisted into adulthood (Yi et al., 2021). Additionally, fetal livers displayed increased OS-related proteins [including NADPH oxidase (NOX)2 (also known as CYBB), NOX4, heme oxygenase-1 (HO-1; also known as HMOX1) and superoxide dismutase (SOD)2)] and SOD activity, and a decrease in glutathione content (Yi et al., 2021). Because OS is a hallmark of metabolic diseases, the markers of OS detected in this study provide evidence that maternal Cd exposure perturbs metabolism during critical windows of liver development. Perinatal Cd exposure has been linked to histological signs of steatosis and fibrosis and increased expression of MASLD-related and imprinted genes, indicating that early-life exposure is sufficient to program MASLD in young animals (Riegl et al., 2023). Another study revealed a sex-specific difference in the effects of maternal Cd exposure on offspring later in life. Although both males and females had dysregulated glucose homeostasis, females, overall, had more severe outcomes, including dyslipidemia, hepatic steatosis, perturbed expression of MASLD-related genes, preneoplastic lesions (Box 2), disrupted retinoic acid signaling, mitochondrial dysfunction, and oxidative and ER stress (Jackson et al., 2020). Finally, the impact of developmental Cd exposure on hepatic glycogen regulation is less clear; one study found that maternally exposed pups had decreased glycogen content in their livers (Pillai and Gupta, 2005), whereas other studies found that liver glycogen content was unaffected by maternal Cd exposure (Roelfzema et al., 1987; Jackson et al., 2020; Yoruk et al., 2003).

Gestational Cd exposure also impacts protein degradation by inducing ubiquitination (Box 2) (Kurita et al., 2016, 2018). One study demonstrated increased abundance of polyubiquitinated protein and decreased monoubiquitinated protein in fetal livers (Kurita et al., 2018). Another showed a decrease in the transcript level of ubiquitin C (*Ubc*), which encodes a protein that maintains stress-related responses and removes damaged or misfolded proteins (Kurita et al., 2016). Because disturbed expression of *Ubc* can lead to defective hepatocyte proliferation (Park et al., 2013), these findings present a possible mechanism through which Cd impacts liver development.

Non-mammalian studies have also shown that developmental Cd exposure adversely impacts liver function. *In ovo* Cd exposure causes significant changes in the size, number and structure of the mitochondria in chicken embryo hepatocytes, which may alter their function and induce OS (Dżugan et al., 2018). Meanwhile, *in ovo* Cd exposure during organogenesis leads to sinusoidal dilation and necrosis of the liver and causes ruptured cellular membranes, irregular chromatin condensation, and damaged or missing organelles in hepatocytes (Venter et al., 2015).

Maternal Cd exposure may have lasting effects on offspring liver through altered hepatic DNA methylation and expression of DNA methyltransferases (Dnmt genes). In maternally exposed males, livers were found to have hypermethylated glucocorticoid receptor gene promoters and increased transcript abundance of *Dnmt3a* in the liver, whereas female mice displayed hypomethylation at the hepatic glucocorticoid receptor gene promoter and decreased *Dnmt3a* expression (Castillo et al., 2012).

### Kidney

In humans, the relationship between developmental Cd exposure and kidney health remains poorly understood, and the few studies that do exist examined childhood and adolescent exposure but not the perinatal period. Once Cd enters the body, the kidneys are the primary organs of Cd accumulation, which persists for decades (Genchi et al., 2020). Small amounts of Cd can be detected in fetal kidney tissue (Lutz et al., 1996); therefore, it is plausible that Cd could exert adverse effects on the developing kidney during gestation and early life. One of the few relevant epidemiological studies estimated dietary Cd intake from 1 to 9 years of age, and, at 9 years, a panel of kidney function biomarkers revealed no association between Cd consumption and kidney function (Rodríguez-López et al., 2020).

Rodent studies provide stronger evidence that perinatal Cd exposure disrupts kidney development and function (Table S12). Notably, a majority of these studies do not extensively examine the mechanisms of developmental Cd-induced kidney toxicity but rather measure markers of renal damage or dysfunction. One study found elevated gene expression of kidney injury molecule-1 (*Kim-1*; also known as *Havcr1*), a marker of damaged proximal tubule cells (Box 2) (Blum et al., 2015). Another found disorganized expression of tight junction proteins claudin-5 and claudin-2 in the glomerulus (Box 2) and proximal tubule (Jacquillet et al., 2007). This disorganization was associated with increased fractional excretion of Na^+^, K^+^, Ca^2+^, Mg^2+^ and PO_4_^3−^, indicating dysfunctional glomerular filtration (Jacquillet et al., 2007). HIF-1 provides a possible mechanism of Cd-induced kidney toxicity, as one study found that gestational Cd exposure reduced the DNA-binding ability of HIF-1 protein in GD21 kidneys, which, if sustained, could harm renal function (Jacobo-Estrada et al., 2018).

There is limited evidence that developmental Cd exposure is able to perturb essential metal homeostasis in the kidneys. Fe and Zn levels were decreased and increased, respectively, in kidneys at postnatal day 21, and the Na/K ratio was decreased in fetal kidneys at GD19 (Mikolić et al., 2016). Patients with chronic kidney disease have essential metal imbalances and are at a greater risk of disease progression if the imbalances are not resolved (Xie et al., 2022), suggesting that Cd-induced changes could exacerbate underlying renal conditions.

## Discussion and conclusions

Parental Cd burden before, during and after conception renders offspring more vulnerable to poor birth outcomes, organ dysfunction and disease. Epidemiological studies, in which existing Cd burden is quantified in maternal biofluid or tissue, cord blood or placenta, implicate Cd in the etiology of small neonatal size and childhood neurobehavioral delays, including poor cognitive performance, social impairments and emotional challenges (Kippler et al., 2012; Röllin et al., 2015; Sioen et al., 2013; Vidal et al., 2015; Zhang et al., 2004). However, epidemiological studies that link developmental Cd exposure with congenital anomalies, cardiovascular and reproductive outcomes, or liver and kidney problems in humans, are rare and have consistently failed to find evidence of teratogenicity (Calle et al., 2015; Pi et al., 2018). It is still uncertain whether humans are vulnerable to developmental Cd-induced changes in the heart, liver, kidneys or gonads, simply because very few studies have incorporated measurement of both Cd and parameters related to these organ systems. Studies of maternal smoking provide opportunities to examine populations with high Cd burden and have been performed in maternal-child cohorts (Drake et al., 2015; Filis et al., 2015; O'Shaughnessy et al., 2011), but these studies cannot distinguish the impact of Cd alone from other harmful substances in tobacco smoke. Additional constraints and variability in epidemiological study findings stem from differences in methods and biological samples used to quantify Cd burden. For example, animal models suggest that hair Cd levels do not accurately reflect internal concentrations of Cd (Combs et al., 1983; Tete et al., 2014). Quantifying Cd levels in newborn tissues may also not accurately reflect maternal exposure owing to placental accumulation of Cd. Opportunities remain to widen the breadth of epidemiological studies that include measurement of maternal Cd burden.

Findings from experimental studies align with neurodevelopmental and growth impairment observations in humans. In contrast, rodent models have yielded evidence that developmental Cd exposure induces congenital anomalies and changes in the cardiovascular and reproductive systems, despite a lack of such evidence from epidemiological studies. Overall, cardiomegaly is the most commonly observed cardiovascular outcome. Although the reproductive organs exhibit cellular and molecular changes, there is little evidence of infertility linked to developmental Cd exposure. Disparate findings between humans and rodents may be explained by differences in anatomy and physiology, route of administration, form of Cd salt or genetic backgrounds. Rats and mice are the only placental species among the commonly used animal models of developmental exposure; however, their placental structures differ from those of humans and may be more susceptible to Cd damage or transport, which could explain why there are more severe outcomes in rodent offspring. Additionally, Cd doses that are much higher than typically encountered in the environment are often used in toxicological studies to elucidate a mechanism of action and may lead to exaggerated or unexpected consequences, a phenomenon particularly noticeable in the context of congenital anomalies (Adami et al., 2011). Humans are exposed to Cd primarily through ingestion or inhalation (WHO, 2019); therefore, Cd administered to animals through injections may not result in similar mechanisms of absorption and action. However, precisely timed injections might be most suitable when investigating specific developmental stages. In aqueous solution, Cd is in the form of a soluble salt that is converted to free ionic Cd, whereas humans ingest ligand-bound Cd. Additionally, gastrointestinal absorption rates differ between species and depend on factors such as Fe intake, making it difficult to convert Cd intake levels in animals to the amount of equivalent human exposure.

In conclusion, the findings of this Review encourage mitigation of parental Cd exposure during the perinatal period. Owing to overwhelming evidence from both controlled experiments and human populations, we conclude that FGR and neurobehavioral impairments are the outcomes of greatest concern. Further studies of the efficacy of intervention with antioxidant and essential trace metals are warranted, and additional recommendations include more robust investigation of the effects of developmental Cd exposure on reproductive, hepatic and renal function through epidemiological studies with longer routine monitoring.

## Supplementary Material

10.1242/dmm.052038_sup1 Supplementary information
