# Supplementary material for: The effects of developmental cadmium exposure on health and disease
Source: Dis Model Mech. 2025 Jun 9;18(6):dmm052038. doi: 10.1242/dmm.052038 (PMC12147461; doi:10.1242/dmm.052038)
Supplement: Supplementary information [file dmm-18-052038-s1.pdf]

Table S1. Key findings related to LBW from select epidemiological studies of maternal Cd burden

Location	Sample Type	Key Findings	Source
Durham County, North Carolina, USA	Maternal blood	Birth:<br>• Reduced infant weight	(Vidal et al., 2015)
Hubei Province, China	Cord blood	Birth:<br>• Reduced infant length<br>• Reduced infant weight	(Tian et al., 2009)
	Maternal blood	Birth:<br>• Reduced infant length	
Hubei Province, China	Cord blood	Birth:<br>• Reduced infant length	(Zhang et al., 2004)
Coastal South Africa	Maternal blood	Birth:<br>• Reduced infant weight	(Röllin et al., 2015)
Wuhan, China	Maternal urine	Birth:<br>• Reduced weight of female infants	(Cheng et al., 2017)
Guangdong Province, China	Maternal urine	Birth:<br>• Reduced weight of female infants<br>• Reduced length of female infants	(Zhang et al., 2018a)
Bristol, England	Maternal blood	Birth:<br>• Reduced weight of female infants<br>• Reduced length of female infants	(Taylor et al., 2016)
Rochester, New York, USA	Maternal urine, placenta	Birth:<br>• No difference in birth weight or length	(Barrett et al., 2023)

Table S2. Key findings related to fetal growth restriction and low birth weight from experimental animal models of early life Cd exposure

Model	Exposure Methods	Key Findings	Source
Wistar rats	10 ppm Cd from cadmium chloride ($CdCl_2$) in maternal drinking water from weaning to mating, 50 ppm Cd during gestation	Postnatal day (PND) 0:<br>• Reduced pup weight	(Castillo et al., 2012)
Wistar rats	3, 15, 30 or 50 ppm Cd from $CdCl_2$ through drinking water during gestation	PND0, 50 ppm:<br>• Reduced pup weight<br>PND0; 3, 15, and 30 ppm Cd:<br>• No difference in pup weight	(Ronco et al., 2009)
Wistar rats	70 mg/L $CdCl_2$ with or without 70 mg/L of $CuSO_4$ in maternal drinking water during gestation	PND0, Cd alone:<br>• Reduced pup weight<br>PND0, Cd with Cu:<br>• No difference in pup weight	(Enli et al., 2010)
Wistar rats	30 ppm Cd from $CdCl_2$ in maternal drinking water during gestation	PND0:<br>• No difference in pup weight<br>• No difference in pup length	(Ronco et al., 2011)
Wistar rats	Maternal subcutaneous (SQ) injection of 0.3 or 0.6 mg/kg Cd from $CdCl_2$ from gestational day (GD) 7-15	PND1, 4, 7, 14, and 21; 0.3 or 0.6 mg/kg:<br>• No difference in pup weight	(Minetti and Reale, 2006)
Sprague-Dawley rats	Maternal intraperitoneal (IP) injection of 0.25 or 0.5 mg/kg Cd from $CdCl_2$ from GD4-19	GD20, 0.5 mg/kg:<br>• Reduced fetal weight<br>GD20, 0.25 mg/kg<br>• No difference in fetal weight	(Zhang et al., 2016b)
C57BL/6 mice	Maternal IP injection of 4 mg/kg $CdCl_2$ on GD8, with or without zinc chloride ($ZnCl_2$)	GD9 and GD11, Cd alone:<br>• Reduced fetal length<br>GD9 and GD11, Cd with Zn:<br>• Fetal length returned to normal	(Fernandez et al., 2003)
C57BL/6 mice	10 mg/L $CdCl_2$ in maternal drinking water during gestation	PND0:<br>• No change in pup weight or length	(Zhao et al., 2018)
C57BL/6J and CAST/EiJ hybrid mice	1 or 50 ppm $CdCl_2$ in maternal drinking water for 5 weeks prior and during gestation	PND0, 1 ppm:<br>• Reduced pup weight in males with CAST/EiJ mothers<br>PND0, 50 ppm:<br>• Reduced pup weight in all groups<br>6 months old, 50 ppm:<br>• Reduced pup weight in females with C57BL/6J mothers	(Hudson et al., 2021)
CD-1 mice	Daily maternal IP injection of 0.5 mg/kg $CdCl_2$ from GD13-17	PND0:<br>• Reduced pup weight<br>• Reduced pup length	(Ji et al., 2011)
CD-1 mice	Maternal inhalation of 100 or 230 μg cadmium oxide (CdO) from 4.5-16.5 DPC	PND0, 100 μg:<br>• No change to pup weight or length<br>e14.5 and 17.5, 230 μg:<br>• Reduced embryo length<br>• No difference in embryo weight<br>PND0-PND21, 230 μg:<br>• Reduced growth rate of neonates	(Blum et al., 2012)
Zebrafish	Aqueous exposure of embryos to 1-200 nM cadmium-telluride (CdTe) quantum dots	120 hours post-fertilization (hpf), 25-200 nM:<br>• Dose-responsive reduction in body length	(Zhang et al., 2012)
Zebrafish	Aqueous exposure of embryos to 0.8-104.1	96 hpf, 17.8 μM:<br>• Reduced body size	(Zhang et al., 2015)

Disease Models & Mechanisms • Supplementary information

	µM Cd as $CdCl_2$ from 0-96 hpf		
Cobb chickens	Shell-less culture with 89 µM Cd from cadmium acetate (CdAc) for 60 hours, with or without zinc acetate (ZnAc)	48 hours after treatment, Cd alone:<br>• Reduced embryo weight<br>48 hours after treatment, Cd with Zn:<br>• No reduction in embryo weight	(Thompson and Bannigan, 2001)

Disease Models & Mechanisms • Supplementary information

Table S3. Key findings related to placenta development and function from epidemiological studies of maternal Cd burden

Population	Sample Type	Key Findings	Source
Matlab, Bangladesh	Placenta, cord blood	Birth:<br>• Elevated placental MT	(Kippler et al., 2012)
Shantou, Guangdong Province, China	Placenta	Birth:<br>• Shortened placental telomere length	(Lin et al., 2013)
New Hampshire, Rhode Island, and Massachusetts, USA	Placenta	Birth:<br>• DNA hypermethylation in placenta<br>• Dysregulated expression of genes and loci related to inflammatory signaling, cell growth, and birth metrics in placenta	(Everson et al., 2018)
Japan	Maternal blood	During pregnancy:<br>• Diagnosis of placenta previa	(Tsuji et al., 2019)
Zagreb, Croatia	Placenta	Birth:<br>• Increased placental Cd accumulation in smokers<br>• Placental insufficiency in smokers<br>• Increased placental Zn in smokers<br>• No effect of smoking status on placental estrogen or progesterone (P4)	(Stasenko et al., 2010)
New Hampshire, Rhode Island, and Massachusetts, USA	Placenta	Birth:<br>• Placental upregulation of five miRNAs related to nervous system development	(Tehrani et al., 2023)

Table S4. Key findings related to placenta development and function from experimental animal models of gestational Cd exposure

Species	Exposure Methods	Key Findings	Source
Wistar-Porton rats	Single maternal intravenous (IV) injection of 1.25 mg/kg Cd on GD20	GD20:<br>• Degeneration of maternal vasculature<br>• Loss of placenta architecture<br>• Clotting and retroplacental hemorrhage	(Samarawickrama and Webb, 1979)
Wistar rats	Maternal SQ injection of 0-1.6 mg/kg $CdCl_2$ daily throughout gestation	GD19, 0.1, 0.4, 0.8, 1.6 mg/kg:<br>• Reduced placental weight:<br>Gd19, 1.6 mg/kg<br>• Increase in collagen fibers in the basal membranes of fetal vasculature	(Hazelhoff Roelfzema et al., 1985)
Wistar rats	Maternal SQ injection of 0.49 mg/kg Cd as $CdCl_2$ daily throughout gestation	PND14-18:<br>• Elevated labyrinth glycogen	(Roelfzema et al., 1987)
Wistar rats	Maternal SQ injection of 0.49 mg/kg Cd as $CdCl_2$ daily throughout gestation	PND18-20:<br>• Elevated labyrinth glycogen<br>PND14-20:<br>• Increasing placental Cd accumulation over time	(Hazelhoff Roelfzema et al., 1987)
Sprague-Dawley rats	Maternal SQ injection of 40 µmol/kg $CdCl_2$ on GD18	GD18:<br>• Ultrastructural cytological changes in the labyrinth<br>• Disturbed maternal and fetal circulation<br>• Slight reduction in placental succinate dehydrogenase activity	(Cho et al., 1988)
Charles Foster rats	SQ injections of 0.05 mg/kg/day CdAc from proestrus through GD5	GD5:<br>• Reduced Zn in MT fraction of the placenta<br>• Reduced placental protein, DNA, RNA, cholesterol, total lipid, and glycogen<br>• Reduced steroidogenic enzymes, P4, and estradiol (E2) in the placenta	(Nampoothiri and Gupta, 2008)
Wistar rats	3-50 ppm Cd^{2+} from $CdCl_2$ in maternal drinking water through GD20	GD20, 50 ppm:<br>• Elevated placental corticosterone<br>GD20, 3-30 ppm:<br>• No difference in placental corticosterone	(Ronco et al., 2009)
Fischer 344 rats	Maternal SQ injection of 0.2 or 2.0 mg/kg/day Cd as $CdCl_2$ from GD11-19	GD20, 2.0 and 0.2 mg/kg/day:<br>• Reduction in placental trophoblast cells<br>• Reduced placenta weight<br>• Down regulation of lactogen genes in placenta<br>GD20, 2.0 mg/kg/day only:<br>• increase in junctional zone apoptosis.<br>No change in spongiotrophoblast and trophoblast giant cells in the placental labyrinth zone at either dose	(Lee et al., 2009)
Wistar rats	70 mg/L $CdCl_2$ in maternal drinking water throughout gestation with or without 70 mg/L $CuSO_4$	GD21, Cd only:<br>• Cd accumulation in the placenta<br>• Increased oxidative stress in the placenta<br>• No change in placenta weight<br>GD21, Cd with Cu:<br>• Alleviation of oxidative stress<br>• No alleviation of placental Cd accumulation	(Enli et al., 2010)
Wistar rats	30 ppm Cd from $CdCl_2$ in maternal drinking water during gestation	GD20:<br>• Increased placental expression of NF-κB	(Ronco et al., 2011)
Sprague-Dawley rats	Maternal IP injection of 0.5 mg/kg Cd from $CdCl_2$ daily from GD5-19	GD20:<br>• Thickened vessel walls<br>• Excessive perivillous fibrin deposition in the labyrinth<br>• Vacuolization and swelling of decidua<br>• Increased placental corticosterone	(Wang et al., 2014)

		• Reduced placental *11β-HSD2* gene expression and protein abundance	
Wistar rats	50 mg/L Cd as $CdCl_2$ in maternal drinking water throughout gestation	GD20:<br>• Elevated placental Zn<br>• No change in placental Fe, Cu, P4 and testosterone (T)	(Mikolić et al., 2015)
Sprague-Dawley rats	1, 3, or 9 mg/kg/day $CdCl_2$ via oral gavage until GD20	GD20:<br>• Altered placental abundance of 15 proteins, including reduced ABCG2 and ABCB4	(Liu et al., 2016)
Wistar rats	SQ 0.04 mmol/kg Cd injection on GD18	6-24 hours post-injection:<br>• Placental necrosis and apoptosis<br>• Cd accumulation in the fetal portion of the placenta	(Yamagishi et al., 2016)
Wistar rats	SQ injection of 0.5 mg/kg/day Cd daily during gestation	GD15, 17, 19, and 21:<br>• Reduced placenta size<br>• Inhibited trophoblast proliferation<br>• Increased apoptosis in trophoblasts	(Erboga and Kanter, 2016)
Sprague-Dawley rats	Maternal IP injection of 0.25 or 0.5 mg/kg $CdCl_2$ from GD4-19	GD20, 0.25 mg/kg:<br>• Increased expression of oxidative stress and antioxidant proteins<br>• Activated DNA repair in placenta<br>• Decreased placental total antioxidant capacity<br>GD20, 0.5 mg/kg:<br>• fetal lethality largely prohibited analysis	(Zhang et al., 2016b)
Wistar rats	Maternal SQ injection of 10 mg/kg Cd from $CdCl_2$ on GD4, 7, 10, or 15	GD20, all treatment timepoints:<br>• Changes in levels of placental glycosylated lectins	(Diaz et al., 2017)
CD-1 mice	Maternal inhalation of 100 or 230 µg CdO from 4.5 to 16.5 DPC	e10.5:<br>• Reduced placenta weight<br>e14.5 and 17.5:<br>• Increased placenta weight	(Blum et al., 2012)
ICR mice	Maternal IP injection of 4.5 mg/kg $CdCl_2$ on GD9 with or without pretreatment with alpha-phenyl-N-t-butylnitrone (PBN), an antioxidant	GD9, Cd alone:<br>• Decrease in placenta weight<br>• Decrease in average blood sinusoid area in the labyrinth<br>• Decreased proliferation and increased apoptosis in the labyrinth<br>• Increased placental endoplasmic reticulum (ER) stress<br>• Increased placental oxidative stress<br>• Decreased placental glutathione (GSH)<br>GD9 Cd with PBN:<br>• Attenuation of decrease of placental GSH<br>• Attenuation of increase in placental oxidative stress and ER stress	(Wang et al., 2012)
CD-1 mice	Maternal IP injection of 3.0 mg/kg $CdCl_2$ on GD15	GD15, 8 hours post-injection:<br>• Upregulation of inflammatory cytokine genes (*Il-1β*, *Mip-2*)<br>GD16:<br>• Upregulation of inflammatory cytokine genes (*Tnf-α, Kc*, and *Mip-2*)<br>• Activation of the Akt pathway	(Hu et al., 2018)
CD-1 mice	Maternal IP injection of 1.0 mg/kg/day $CdCl_2$ from GD13-17 with or without co-injection of N-acetylcysteine (NAC)	GD18, Cd alone:<br>• Decreased placental weight<br>• Decreased internal space of blood vessels in the labyrinth<br>• Reduced cell proliferation<br>• ER stress<br>• Reduced expression of growth factors and nutrient transporters<br>GD18, Cd with NAC:<br>• Attenuation of placental effects	(Guo et al., 2018)

Disease Models & Mechanisms • Supplementary information

C57BL/6J	1 or 50 ppm $CdCl_2$ in maternal drinking water before and during gestation	e18.5, 50 ppm:<br>• Decreased relative female placenta weight<br>• Increased expression of imprinted growth regulation gene *Cdkn1c*<br>• Increased labyrinth area fraction<br>e16.5, 50 ppm:<br>• Decreased raw male placenta weight<br>• Increased relative male placenta weight	(Simmers et al., 2023)
CD-1	50 or 150 mg/L Cd as $CdCl_2$ in maternal drinking water from GD8-17	GD18<br>• Inhibited placental estrogen synthesis<br>• Reduced placental E2<br>• Downregulation of estrogen synthases CYP17A1 and CYP19	(Liu et al., 2022b)

Disease Models & Mechanisms • Supplementary information

Table S5. Key findings from *in vitro* studies of the effect of Cd exposure on the placenta

Tissue/cell type	Exposure Methods	Key Findings	Source
Placental lobules from healthy, nonsmoking women immediately after birth	Perfusion for 4 hours with 10, 20 or 100 nmol/mL Cd as $CdCl_2$ with or without $ZnCl_2$	10nmol/mL:<br>• Reduced placental transfer of Zn<br>20 and 100 nmol/mL<br>• Loss of volume in fetal-origin placental vasculature<br>100 nmol/mL:<br>• Placental necrosis<br>No significant differences in oxygen consumption, lactate production, glucose consumption, or amino acid uptake for all doses	(Wier et al., 1990)
Primary human trophoblasts	Cultured for 96 hours with 5, 10, or 20 μM $CdCl_2$	10 and 20 μM:<br>• Decrease in leptin transcript abundance	(Stasenko et al., 2010)
JEG-3, hypertriploid human trophoblast cell line	Cultured for 48 hours with 0.028, 1, 10, or 25 μM $CdCl_2$	1-25 μM:<br>• Impaired cell migration<br>0.028 and 1 μM:<br>• Upregulation of *TGFB1* gene expression<br>10 and 25 μM:<br>• Downregulation of *TGFB1* gene expression<br>• Downregulation of *TGFB1R* gene expression<br>• Downregulation of *SMAD2* gene expression<br>• Upregulation of *SMAD3* gene expression	(Brooks and Fry, 2017)
HTR-8/SVneo, human trophoblast cell line	Cultured for 24 hours with 2 or 20 μM $CdCl_2$ with or without overexpression of S100P	2 and 20 μM Cd alone:<br>• Inhibited cell proliferation<br>• Decreased gene and protein abundance of Ca^{2+} transporter S100P<br>With over-expression of S100P:<br>• Attenuation of Cd-induced reduction in cell proliferation	(Zhou et al., 2016)
JEG-3, hypertriploid human trophoblast cell line	Cultured for 6 hours with 0, 3.125, 6.25, 12.5, 25 or 50 μM $CdCl_2$ or with 25 μM $CdCl_2$ for 2, 6, 12 or 24 h	25 μM of $CdCl_2$ for 24 hours:<br>• Upregulation of inflammatory cytokine genes (*IL8*, *IL6*, *TNFA*)<br>• Activation of Akt pathway	(Hu et al., 2018)
BeWo, human epithelial choriocarcinoma cell line	Cultured for 24 hours with 2, 5, 10, or 20 μM Cd as $CdCl_2$ with forskolin (FSK), a differentiation inducer	24 hours:<br>• Dose-responsive inhibition of FSK-induced differentiation	(Ogushi et al., 2022)

Disease Models & Mechanisms • Supplementary information

Table S6. Key findings related to congenital anomalies from experimental animal models of early life Cd exposure

Species	Exposure Methods	Key Findings	Source
Wistar rats	Single maternal IV injection of 1.25 mg/kg Cd from GD9-15	GD20:<br>• Hydrocephalus<br>• Anophthalmia<br>• Microphthalmia<br>• Gastroschisis<br>• Umbilical hernia	(Samarawickrama and Webb, 1979)
Albino rats	100 ppm Cd in maternal drinking water from GD0-20	GD20:<br>• No congenital anomalies observed	(Saxena et al., 1986)
Wistar rats	Maternal oral gavage of 2-20 mg/kg Cd from GD7-16	GD21<br>• No congenital anomalies observed	(Barański et al., 1982)
Sprague-Dawley rats	Maternal oral gavage of 10-50 mg/kg Cd from GD6-18	GD19<br>• No congenital anomalies observed	(Wardell et al., 1982)
Wistar rats	Single maternal IV or IP injection of 0.25-1.0 mg/kg MT-bound Cd between GD8-14	GD20:<br>• Hydrocephalus<br>• Urogenital defects	(Webb et al., 1988)
Wistar-Porton rats	Single maternal IV or IP injection of 1.25 mg/kg Cd as $CdCl_2$ on GD8, 10, 12, or 14	GD20, all dosing timepoints:<br>• Higher incidence of malformed fetuses	(Holt and Webb, 1987)
ICR mice	Maternal IP injection of 1 or 2 mg/kg Cd sulfate on GD7	GD18:<br>• Skeletal malformation<br>• External malformations	(Murata et al., 1993)
C57BL/6 mice	Single maternal IP injection of 4.0 mg/kg $CdCl_2$ at 8 DPC	e9 and e11<br>• neural tube defects (open neural tube, midbrain, and hindbrain)	(Fernandez et al., 2003)
C57BL/6J mice	Single maternal IP injection of 4 mg/kg $CdCl_2$ on GD7, 8, 9, or 10	e18.5:<br>• Exencephaly only when dosed on GD7 or 8<br>• Primarily limb defects, but no central nervous system defects when dosed on GD9 or 10<br>• Micropthalmia<br>• Omphalocoele<br>• Heart abnormalities<br>• Tail abnormalities<br>• Face abnormalities	(Webster and Messerle, 1980)
MFI mice	Maternal injection of 4 or 6 mg/kg $CdCl_2$ on GD7	GD18:<br>• Exencephaly<br>• Abnormalities of the ear<br>• Exophthalmia and microphthalmia<br>• Mandibular/maxillary hypoplasia<br>• Edema<br>• Abnormalities of limbs and tail	(Padmanabhan, 1987)
C57BL/6N and SWV/Fnn mice	Maternal IP injection of 4.0 mg/kg $CdCl_2$ on GD9	GD10:<br>• Right-sided preference of forelimb ectrodactyly in C57BL/6N embryos<br>• No anomaly in SWV/Fnn embryos	(Chen et al., 2008)
C57BL/6J mice	Maternal IP injection of 4 mg/kg at e9.0	e18.0:<br>• Right-sided preference of forelimb ectrodactyly	(Elsaid et al., 2010)

Disease Models & Mechanisms • Supplementary information

Zebrafish	Aqueous exposure to 100 μM Cd from 4-24 hpf	24 hpf:<br>• Microphthalmia	(Chow et al., 2009)
Zebrafish	Aqueous exposure to 0.8-104.1 μM from 0-72 hpf	72 hpf, 17.8 μM:<br>• Trunk abnormalities<br>• Hypopigmentation<br>• Head hypoplasia<br>• Microphthalmia<br>• Reduced interorbital distance<br>96 hpf, 17.8 μM:<br>• Trunk abnormalities<br>• Hypopigmentation<br>• Reduced interorbital distance	(Zhang et al., 2015)
Zebrafish	Aqueous exposure to 100 μM Cd from 5 hpf to 24, 48, 72, or 96 hpf, with or without co-exposure to taxifolin, a bioflavenoid	Cd alone, at all timepoints:<br>• Yolk sac edema<br>• Eye defect<br>• Head defect<br>• Sacculi/otoliths defect<br>• Tail curvature defect<br>• Tail tip defect<br>With taxifolin:<br>• All defects abolished	(Manigandan et al., 2015)
Zebrafish	Aqueous exposure 0.5 μM Cd a $CdCl_2$ from 4-96 hpf	96 hpf:<br>• No morphological defects or change in body length	(Di Paola et al., 2022)
Zebrafish	Aqueous exposure to 0.5-20 mg/L CdTe dots from 4-96 hpf	24-96 hpf, 0.5-20 mg/L:<br>• Increase in spinal deformations, dependent on conjugate	(Bosch et al., 2023)
Zebrafish	Aqueous exposure to 183.31 μg/L cd as $CdCl_2$ with or without $ZnCl_2$ from 0-96 hpf	96 hpf, Cd alone:<br>• Pericardial edema<br>• Yolk sac edema<br>• Tail curvature<br>• Crooked bodies<br>• Skeletal malformations (kyphosis, lordosis, and scoliosis<br>96 hfp, with Zn:<br>• Partial or total correction of anomolies	(Chouchene et al., 2022)
Cobb chickens	Shell-less culture with 89 μM Cd^{2+} from CdAc for 60 hours, with or without ZnAc	48 hours post-treatment, Cd alone:<br>• Limb abnormalities<br>• Anterior body wall defects<br>• Abnormalities of truncal curvature<br>• Defects of cranial aspect of neural tube<br>• Eye abnormalities<br>• Facial abnormalities<br>48 hours post-treatment Cd with Zn:<br>• Reduced malformation frequency	(Thompson and Bannigan, 2001)

Disease Models & Mechanisms • Supplementary information

Table S7. Key findings related to cardiovascular development and disease from experimental animal models of early life Cd exposure

Species	Exposure Methods	Key Findings	Source
Long Evans rats	SQ injection of 10 µmol/kg Cd at PND9	PND20:<br>• Decreased cardiac Zn<br>PND36:<br>• Increased cardiac Zn<br>• Increased cardiac Cu	(Thomas and Mushak, 1986)
Wistar rats	75 ppm Cd in maternal and paternal drinking water for two months prior to mating and during gestation	PND7:<br>• Increased cardiac lipid peroxidation	(Xu et al., 1993)
Wistar rats	50 ppm Cd in maternal drinking water throughout gestation	PND42:<br>• Increase in overall cardiac RNA synthesis<br>• No significant differences in cardiac muscle protein synthesis	(Konecki et al., 2003)
Wistar rats	30 ppm Cd in maternal drinking water throughout gestation	PND60-70:<br>• Decreased endothelium-dependent reactivity in aortic rings<br>• Increased thickness of aortic and anterior left ventricular walls<br>• Increased aortic levels of HO-1 that was more prominent in females	(Ronco et al., 2011)
Wistar rats	30 ppm Cd in maternal drinking water throughout gestation	PND60-70:<br>• reduced ischemia-induced cardiac injury during myocardial infarction	(Zepeda et al., 2012)
BALB/c mice	Maternal IP injection of 2.5 or 4.5 mg/kg Cd on GD9 with or without co-exposure to 6-Formylindolo[3,2-b]carbazole (FICZ, an endogenous activator of AHR)	GD18, 2.5 mg/kg and 4.5 mg/kg Cd alone:<br>• Cardiac hypertrophy<br>• Increased cardiac connective tissue<br>• Increased mean cardiomyocyte volumes<br>• Increased cardiac expression of AHR-Wnt/β-catenin<br>GD18, 4.5 mg/kg Cd alone:<br>• Increased length of microvessels<br>• Decreased volume of cardiomyocytes and cardiac vessels<br>GD18 2.5 mg/kg and 4.5 mg/kg Cd with FICZ:<br>• Abnormalities were exacerbated	(Omidi et al., 2019)
C57BL/6J mice	0.5 or 5 ppm Cd in drinking water from conception to 13 weeks of age	PND90, 0.5 and 5 ppm:<br>• No induction of cardiac hypertrophy or fibrosis	(Liang et al., 2019)
C57BL/6J and CAST/EiJ hybrid mice	1 or 50 ppm $CdCl_2$ in maternal drinking water for 5 weeks prior and during gestation	PND0, 1 ppm:<br>• Female cardiac hypertrophy<br>PND0, 50 ppm:<br>• Cardiac hypertrophy<br>• Differentially expressed genes relevant to enlarged hearts, hypertension, abnormal heart and cardiovascular system development, nutritional disease, hypoxia, cellular energy and carbon metabolism, ROS, nitric acid homeostasis and metal homeostasis<br>6 months old, 50 ppm:<br>• Hypertension<br>• Increased tail blood volume	(Hudson et al., 2019)
Zebrafish	Aqueous exposure to 100 µM Cd from 5-48 hpf	48 hpf:<br>• Localized vascular defects in dorsal aortae, segmental, and cranial vessels<br>• Reduced complexity of the craniofacial vasculature	(Cheng et al., 2001)

Disease Models & Mechanisms • Supplementary information

Zebrafish	Aqueous exposure to 25-300 µM Cd as CdTe dots from 6-48 hpf	48 hpf:<br>• Reduced heart rate<br>96 hpf:<br>• Vascular hyperplasia<br>• Vascular crossing<br>• Vascular turbulence<br>• Vascular bifurcation	(Zhang et al., 2012)
Zebrafish	Aqueous exposure to 0.01-10 µM Cd from 24-96 hpf	120 hpf, 0.01-10 µM:<br>• Dose-responsive increase in heart rate	(Wold et al., 2017)
Japanese medaka fish	Exposure to sediment with 0.3x, 1x, and 3x of their environmental habitat's Cd concentrations	168 hpf, 1x and 3x:<br>• Increased heart rates<br>PND0, 0.3x and 3x:<br>• Abnormal heart positioning<br>• Heart looping	(Barjhoux et al., 2012)
American bullfrog	Aqueous exposure to 1 ppb Cd for 2 or 16 days	PND7:<br>• Loss of cardiac contractility, more pronounced with longer exposure	(Dal-Medico et al., 2014)

Disease Models & Mechanisms • Supplementary information

Table S8. Key findings related to neurodevelopment and behavior from epidemiological studies of maternal Cd burden

Population	Sample Type	Key Findings	Source
Haguenau, France	Maternal hair	6 years old:<br>• Impaired general cognitive, perceptual, quantitative, and motor skills	(Bonithon-Kopp et al., 1986)
	Newborn hair	6 years old:<br>• Impaired perceptual and motor skills	
Hubei Province, China	Cord blood	4.5 years old:<br>• Lower IQ	(Tian et al., 2009)
Matlab, Bangladesh	Maternal urine	5 years old:<br>• Lower IQ scores, more pronounced effect in female offspring	(Kippler et al., 2012)
Flanders, Belgium	Cord blood	7-8 years old:<br>• Higher risk of emotional problems in boys only	(Sioen et al., 2013)
South Korea	Maternal blood	5 years old:<br>• Lower performance IQ<br>• No difference in cognitive IQ	(Jeong et al., 2015)
Crete, Greece	Maternal urine	4-5 years old:<br>• Lower general cognitive function	(Kippler et al., 2016)
Shandong, China	Maternal blood	12 months old:<br>• Lower infant social domain developmental quotient	(Wang et al., 2016a)
Spain	Placenta	4-5 years old:<br>• No difference in general cognitive function	(Freire et al., 2018)
Japan	Maternal Blood	0.5-1.5 years old:<br>• Impaired gross motor function<br>• Impaired problem solving<br>2-3 years old<br>• All impairments resolved	(Masumoto et al., 2022)
Guanxi, China	Maternal serum	2-3 years:<br>• Impaired gross motor function	(Liu et al., 2022a)
	Infant urine	2-3 years:<br>• Impaired language development	
Rio de Janeiro, Brazil	Maternal blood	6 months:<br>• No impairment in social, language, or motor skills	(de Assis Araujo et al., 2022)
Poland	Cord blood	1-2 and 7 years old<br>• No impairment in behavior or cognition	(Garí et al., 2022)
Rhode Island, New Hampshire, and Massachusetts, USA	Placenta	Birth:<br>• Upregulated placental miRNAs related to nervous system development<br>• Decrease in infant quality of movement and increase in excitability	(Tehrani et al., 2023)

Disease Models & Mechanisms • Supplementary information

Table S9. Key findings related to neurodevelopment and behavior from experimental animal models of early life Cd exposure

Species	Exposure Methods	Key Findings	Source
Holtzman rats	Maternal IP injection of 2.0 mg/kg Cd on GD20	GD21<br>• Formation of endothelial cell vacuoles in brain capillaries	(Rohrer et al., 1978)
Wistar rats	Maternal oral gavage of 0.4 or 4 mg/kg Cd before and during gestation	PND60, 0.4 or 4 mg/kg:<br>• Reduced exploratory locomotor activity	(Barański et al., 1983)
Sprague-Dawley rats	Maternal oral gavage of 25 mg/kg/day Cd from GD6-18	PND35-56:<br>• Reduced sorbitol dehydrogenase activity in the brain	(Stewart et al., 1984)
Wistar rats	50 ppm Cd in maternal drinking water throughout gestation	GD20:<br>• Decreased brain Cu<br>• No changes in brain Zn or Fe	(Sowa and Steibert, 1985)
Long Evans rats	SQ injection of 2 or 10 µmol/kg $CdCl_2$ on PND9	PND20, 2 µmol/kg:<br>• Decreased cerebral Cu<br>• Increased cerebellar Cu<br>PND20, 10 µmol/kg:<br>• Decreased cerebral Zn<br>• Decreased cerebellar Zn<br>• Increased cerebellar Cu<br>PND36, 10 µmol/kg:<br>• Increased cerebral Cu<br>• Increased cerebellar Cu	(Thomas and Mushak, 1986)
Wistar rats	4.2 or 8.4 µg/ml Cd in maternal drinking water throughout gestation	PND3-12, 4.2 and 8.4 µg/ml:<br>• Delay in cliff aversion response<br>• Delayed development of straight-line swimming<br>• No difference in surface and air righting and visual placing<br>PND14-21, 4.2 and 8.4 µg/ml:<br>• Increased spontaneous locomotor activity<br>PND60, 4.2 and 8.4 µg/ml:<br>• Decreased spontaneous locomotor activity	(Ali et al., 1986)
Wistar rats	60 ppm Cd in maternal drinking water throughout gestation	PND14:<br>• Decreased brain Cu<br>PND70-154:<br>• Decreased exploratory locomotor activity<br>• Decreased brain Zn<br>• Decreased grooming behavior in males<br>• Decreased avoidance behavior in females	(Barański, 1986)
Wistar rats	60 or 180 ppm Cd in maternal drinking water, GD1-20	GD20, 60 ppm:<br>• Decreased fetal brain Zn<br>• No difference in fetal brain Cu<br>GD20, 180 ppm:<br>• No difference in fetal brain Cu or Zn	(Barański, 1987)
CFY rats	Daily maternal SQ injection of 0.2, 0.62, 2.0 mg/kg Cd from GD7-15	PND38-42; 0.62 and 2.0 mg/kg:<br>• Reduced horizontal motor activity<br>• Delayed acquisition and extinction of the conditioned escape response<br>• Increased time spent in immobility during a swim stress test<br>PND90-100; 0.2, 0.62, and 2.0 mg/kg:<br>• Reduced horizontal motor activity<br>PND90-100; 0.62 and 2.0 mg/kg:<br>• Delayed acquisition and extinction of the conditioned escape response<br>• Increased aggression	(Lehotzky et al., 1990)

Disease Models & Mechanisms • Supplementary information

Albino Druckrey rats	50 ppm Cd in maternal drinking water throughout gestation	PND7-14:<br>• reduced brain weights<br>• reduced activity of mitochondrial enzymes<br>PND21::<br>• No difference in brain weights<br>• Reduced activity of mitochondrial enzymes	(Gupta et al., 1991)
Wistar rats	30 or 75 ppm Cd in paternal and maternal drinking water for two months before and throughout gestation	PND7, 30 and 75 ppm:<br>• No change in brain weight<br>PND7, 75 ppm only:<br>• Increased lipid peroxidation in the brain	(Xu et al., 1993)
Albino Druckrey rats	20 ppm Cd in maternal drinking water during gestation and lactation	PND1-21:<br>• Cd accumulation in brain<br>• No difference in brain weight<br>• Increased lipid peroxidation in brain<br>• Increased brain TSH<br>• Increased brain catalase activity<br>• Increased GSH reductase activity<br>PND1 only:<br>• Reduced brain GSH<br>• Increased brain superoxide dismutase (SOD) activity<br>• Increased GSH peroxidase (GPx) activity<br>PND7-21:<br>• Increased brain GSH<br>• Reduced brain SOD activity<br>• Reduced brain GSH peroxidase activity	(Gupta et al., 1995)
Albino Druckrey rats	20 ppm Cd in maternal drinking water during gestation	PND1-21<br>• Increased brain SOD activity<br>• Increased brain catalase activity<br>• Increased brain GSH reductase activity<br>GD18 and GD 20<br>• Decreased fetal brain GSH peroxidase	(Gupta et al., 1996)
Albino Druckrey rats	10 ppm Cd in maternal drinking water during gestation, lactation, and up to PND45 in offspring water	PND15-45:<br>• No change in brain weight<br>• Reduced total brain lipids<br>• Reduced brain cholesterol<br>• Increased brain phosphatidylethanolamine<br>PND15 only:<br>• Increased total brain triglycerides<br>PND15-21:<br>• Reduced brain Zn and Cu<br>PND21-45:<br>• Reduced total brain gangliosides<br>• Reduced brains galactolipids<br>• Altered brain phosphatidylcholine<br>PND45 only:<br>• Reduced brain phosphatidylcholine, phosphatidylserine, and sphingomyelin	(Gupta and Shukla, 1996)
Wistar rats	Maternal oral treatment with 3.5, 7.0 or 14.0 mg/kg Cd from $CdCl_2$ during mating, gestation and lactation, with or without continued treatment and mating of the 2nd generation	2nd generation and 3rd generation, 3.5, 7.0 or 14.0 mg/kg:<br>• Dose-responsive increase in ECoG mean frequency and evoked potential latency in the somatosensory, visual, and auditory foci of the brain<br>• Dose-responsive increase in the refractory period of the tail nerve<br>• Dose-responsive decrease in the ordinate-conduction velocity of the tail nerve<br>• Dose-responsive decrease in ECoG index in the somatosensory, visual, and auditory foci of the brain	(Nagymajtényi et al., 1997)
Swiss albino rats	15 ppm Cd from $CdCl_2$ in maternal drinking water during gestation,	PND60, with postnatal exposure:<br>• Impaired visual evoked potentials	(Yargiçoğlu et al., 1997)

Disease Models & Mechanisms • Supplementary information

	with or without continued exposure until PND60	• Increased lipid peroxidation in the brain<br>PND60, without postnatal exposure:<br>• Visual evoked potential and lipid peroxidation parameters largely rescued	
Sprague-Dawley rats	5 ppm Cd in maternal drinking water from PND0-19, with or without continued exposure until PND42	PND42, with or without post-weaning Cd:<br>• No detected Cd in brain<br>• No change in brain weight<br>PND42, without post-weaning Cd:<br>• Reduced cortical hippocampal levels of 5-hydroxyindoleacetic acid compared to those without lactational exposure<br>• Reduced cortical levels of serotonin compared to those without lactational exposure	(Andersson et al., 1997)
Inbred albino Wistar rats	10 mg/L CdAc in maternal drinking water during gestation, with or without exposure until PND5	PND0:<br>• Increased raw brain weight<br>PND5:<br>• Increased raw and proportional brain weight<br>• Reduced brain nucleic acid content	(Antonio et al., 1998)
Wistar rats	Daily IP injections of 1 mg/kg Cd to PND13-17 pups, with or without dexamethasone, an MT inducer	PND18, Cd without dexamethasone:<br>• Increased striatal levels of Cd and MT<br>• Increased K+-evoked dopamine release from striatum<br>• Inhibited striatal tyrosine hydroxylase (TH) activity<br>• No differences in striatal homovanillic acid or dopamine levels when measured alone, but a significant increase in their ratio (HVA/DA)<br>• No difference in monoamine oxidase activity<br>• Increased striatal lipid peroxidation<br>PND18, Cd with dexamethasone:<br>• TH activity and HVA/DA ratio rescued	(Gutiérrez-Reyes et al., 1998)
Wistar rats	0.5, 7 or 14 mg/kg oral Cd treatment from $CdCl_2$ on either: GD5-15, GD5-15 + 4 weeks of lactation, GD5-15 + 4 weeks of lactation + 8 weeks offspring treatment	PND84; 7 and 14 mg/kg, GD5-15 and lactation<br>• reduced horizonal open field ambulation<br>• reduced rearing activity<br>• reduced exploration of open field center<br>PND84; 7 and 14 mg/kg, gestation, lactation<br>• altered electrophysiological findings	(Dési et al., 1998)
Swiss albino rats	15 ppm Cd in maternal drinking water during gestation only, gestation and lactation until PND22, or gestation, lactation, and in offspring drinking water until PND60	PND60, without post-weaning Cd<br>• Brain Cd accumulation<br>• Brain lipid peroxidation<br>PND60, with post-weaning Cd:<br>• altered electrophysiological features, including slowing of peripheral conduction velocity	(Agar et al., 2000)
Wistar rats	50 ppm Cd in maternal drinking water during gestation	PND42:<br>• Increased overall RNA synthesis in the offspring brain<br>• No differences in brain protein synthesis	(Konecki et al., 2003)
Sprague-Dawley rats	5 or 25 ppm Cd as $CdCl_2$ in maternal drinking water	PND38-42, 5 and 25 ppm<br>• Increased ambulation in a motor activity test<br>PND38-42, 25 ppm<br>• Increased small movements, rearing, and total activity in a motor activity test<br>• No change in brain Zn, learning, or memory	(Grawé et al., 2004)
Wistar rats	Maternal SQ injection with 0.3 or 0.6 mg/kg Cd from $CdCl_2$ from GD7-15	PND3-7, 0.6 mg/kg<br>• Delayed righting reflex<br>• Delayed cliff aversion	(Minetti and Reale, 2006)
Sprague-Dawley rats	10 mg/L Cd from CdAc during gestation and lactation	PND0:<br>• Increased proportional brain weight<br>• Decreased brain SOD activity<br>• Increased brain malondialdehyde (MDA)	(Zhang et al., 2009)

		PND21:<br>• Increased raw and proportional brain weight<br>• Decreased brain GPx activity<br>• Decreased brain SOD activity<br>• Increased brain MDA<br>• Cd accumulation in the brain<br>• Structural brain damage including mitochondrial damage and membrane disorganization	
Wistar rats	Oral gavage of 0.98 $CdCl_2$ from GD5-PND60	PND16:<br>• Cd accumulation in the brain<br>PND60<br>• Cd accumulation in the brain<br>• Hyperactivity<br>• Increased grip strength<br>• Learning-memory deficit<br>• Multiple impaired features of astrocytes	(Rai et al., 2010)
Wistar rats	10 mg/L Cd as CdAc in maternal drinking water during pregnancy and lactation	PND21<br>• Reduced hippocampal levels of serotonin and 5-hydroxyindolacetic acid<br>• Decreased glutamate in the hypothalamus<br>• Increased glutamate in the hippocampus<br>• Decreased GABA in the cerebral cortex	(Antonio et al., 2010)
Norway rats	Maternal SQ injections of 1 or 2 mg/kg Cd as $CdCl_2$ on GD6 with or without co-administration of Se	PND21, 1 and 2 mg/kg Cd alone<br>• Dose-responsive increase in cerebellar Cd<br>• Altered histological features in the cerebellum and dose-dependent increases of Cd in the cerebellum<br>• thickening of cerebellar external granule cell layer<br>PND21, 1 and 2 mg/kg Cd with Se<br>• Attenuation of thickening from 1 mg/kg Cd, but not 2 mg/kg Cd	(Bekheet, 2011)
Wistar rats	Oral gavage of 2.94 ppm $CdCl_2$ from GD5-PND60	PND24-60<br>Reduced abundance myelin-related proteins, including:<br>• Myelin basic protein (MBP)<br>• Myelin proteolipid (PLP)<br>• Cyclin nucleotide phosphodiesterase (CNPase)<br>• Myelin associated glycoprotein (MAG)<br>• Neurofilament (NF)	(Rai et al., 2013)
Wistar rats	50 ppm Cd as $CdCl_2$ in maternal drinking water during gestation and lactation	PND21<br>• Reduced cerebellar acetylcholinesterase (AChE) activity<br>• Increased frontal cortex AChE activity<br>• Increased cerebellar Na^+K^+ATPase activity<br>• Decreased frontal cortex Na^+K^+ATPase activity<br>• Decreased hippocampal Mg^{2+}ATPase activity	(Stolakis et al., 2013)
Wistar rats	50 ppm Cd as $CdCl_2$ in maternal drinking water during gestation with or without continued exposure during lactation	PND0<br>• Increased brain AChE activity<br>• Increased brain Na+,K+-ATPase activity<br>• No difference in brain Mg^{2+}ATPase<br>PND21, with exposure during lactation<br>• Reduced brain AChE activity<br>• No difference in brain Na+,K+-ATPase activity<br>• No difference in brain Mg^{2+}ATPase	(Liapi et al., 2013)
Sprague-Dawley rats	Paternal oral gavage with 22.15mg/kg $CdCl_2$ every 2 days for 9 weeks before mating	PND2-15<br>• Longer surface-righting time<br>• Longer cliff avoidance time<br>• Longer negative geotaxis response time<br>• Shorter forelimb hanging time<br>PND70	(Zhao et al., 2015)

Disease Models & Mechanisms • Supplementary information

		• Reduced abundance of GSH and SOD	
Wistar rats	50 ppm Cd in maternal drinking water from 4 weeks before mating through lactation	PND0:<br>• Reduced brain Fe<br>PND11:<br>• Reduced brain Zn<br>PND21:<br>• Reduced brain Fe<br>PND49:<br>• Reduced brain Fe<br>• Increased brain Zn<br>Cross fostering to control dams rescued brain Fe<br>No difference in raw or proportional brain weights at any time	(Mikolić et al., 2016)
Wistar rats	50 mg/L Cd as $CdCl_2$ in maternal drinking water during gestation and lactation with or without $ZnCl_2$	PND21, Cd without Zn:<br>• Cd accumulation in the brain<br>• Reduced brain Zn<br>• Reduced brain SOD activity<br>• Increased brain MT<br>• Reduced brain gene expression of *Bdnf*<br>• Reduced brain gene expression of Zn transporters<br>• Delayed cliff avoidance response<br>• No difference in behavioral response to the open field test, suspension test, righting reflex, or negative geotoxis test<br>PND21, Cd with Zn:<br>• All outcomes except cliff avoidance response were rescued by Zn co-exposure	(Mimouna et al., 2018)
Wistar rats	50 mg/L Cd as $CdCl_2$ in maternal drinking water during gestation and lactation with or without $ZnCl_2$	PND35 Cd alone:<br>• Detecteable Cd in the brain<br>• Reuced Zn in the brain<br>• Decreased volume of hippocampus and dentate gyrus<br>• Pyknosis in the hippocampus and dentate gyrus<br>• Decreased SOD activity in the brain<br>• Elevated brain MT levels<br>PND35 Cd with Zn<br>• Partial rescue of brain Cd, Zn, and SOD activity<br>• Rescue of hippocampal volume loss and pyknosis	(Ben Mimouna et al., 2018b)
Wistar rats	50 mg/L Cd as $CdCl_2$ in maternal drinking water during gestation and lactation with or without $ZnCl_2$	GD20, without Zn<br>• No detectable Cd in the brain<br>• Decreased brain Zn<br>• No significant differences in relative brain weights<br>• Cortical and hippocampal pyknosis<br>• Increased brain SOD activity<br>• Increased brain MT<br>• Cd altered Zn transporter gene expression<br>• Cd reduced *Bdnf* expression<br>• Increased gene expression of *Mtf1*<br>• No difference in gene expression of two Zn finger proteins (*Sp1*, *Znf536*)<br>GD20 with Zn<br>• SOD activity, Zn transporter gene expression and *Bdnf* gene expression were rescued by Zn co-exposure<br>• Pyknosis, MT, and *Mtf1* gene expression were not rescued	(Ben Mimouna et al., 2018a)
Sprague-Dawley rats	Maternal oral exposure to 1 or 5 mg/kg Cd during gestation and lactation	PND21, 1 and 5 mg/kg<br>• Detectable Cd in the cerebral cortex, but not the hippocampus	(Feng et al., 2019)

Disease Models & Mechanisms • Supplementary information

		• Decreased postsynaptic density thickness and increased synapse cleft<br>PND35, 1 and 5 mg/kg<br>• Increased escape latency in the Morris water maze (MWM) test<br>• Decreased postsynaptic density thickness<br>PND56, 1 and 5 mg/kg<br>• Impaired response to spatial probe test in MWM<br>• Decreased postsynaptic density thickness and increased synapse cleft	
CD-1 mice	50 or 150 mg/L Cd as $CdCl_2$ in maternal drinking water from GD8-17with or without NAC or E2 supplementation	PND140, 150 mg/L, without supplementation:<br>• Longer escape latency<br>PND140, 150 mg/L, with NAC:<br>• Recovery of escape latency<br>GD18, 50 and 150 mg/L, without supplementation:<br>• Disrupted estrogen signaling in fetal brain, including reduced E2 and estrogen receptor abundance<br>• Reduced bone-derived neurotropic factor (BDNF)<br>GD18, 150 mg/L only, without supplementation:<br>• Impaired neuronal development, including reduced postsynaptic density protein 95 (PSD-95) and Synapsin-1 abundance<br>GD18 with E2:<br>• Full or partial recovery of estrogen signaling and neuronal development	(Liu et al., 2022b)
129/Ola and C57BL/6 mice,<br>wild type and MT-null	10 mg/L $CdCl_2$ in maternal drinking water from GD0-PND10	PND10<br>• Reduced serum thyroid hormone thyroxine (T4) in both WT and KO mice<br>• Increased brain type 2 deiodinase activity in KO, not WT<br>• No effect on brain type 3 deiodinase in either WT or KO	(Mori et al., 2006)
C57BL/6J Jcl mice	10 ppm $CdCl_2$ in maternal drinking water from GD0-PND10	PND10<br>• Reduced gene expression of *Pgr* and *ER-α* in female brain<br>• Reduced gene expression of *ER-β* in male brain<br>PND56<br>• No differences in open field behavior	(Ishitobi et al., 2007)
Swiss albino mice	3 ppm Cd through drinking water from GD0-PND5, with or without melatonin	PND6<br>• Increased brain lipid peroxidation and ROS<br>• Increased hippocampal Cd and Fe<br>• Decreased antioxidant activity and AChE content and activity<br>• Increased hippocampal GSH, Cu and Zn<br>Maternal co-exposure to melatonin partially or fully rescued Cd-induced effects	(Mukherjee et al., 2010)
C57BL/6J mice	10 ppm Cd in maternal drinking water from GD1-PND10	PND10<br>• Increased transferrin receptor gene expression in the brain	(Honda et al., 2013)
Swiss albino mice	Maternal IP injection of 1.2 mg/kg Cd daily on PND1-7, with or without quercetin, an antioxidant	Adulthood<br>• Impairment of memory<br>• Increased escape latency<br>• Increased oxidative stress in the brain<br>Maternal co-exposure to quercetin rescued effect of Cd	(Halder et al., 2016)
Albino mice	Maternal IP injection of 10 mg/kg Cd on GD7, PND1, and PND15, with or without co-exposure to parsley juice	PND30<br>• Increased activity<br>• Reduced grip strength and balance<br>• Reduced brain tissue levels of neurotransmitters (dopamine, serotonin, acetylcholine)	(Allam et al., 2016)

Disease Models & Mechanisms • Supplementary information

		• Altered oxidative stress parameters in the brain,<br>• Chromatolysis and pyknosis in pyramidal neurons<br>• Degenerated Purkinje neurons<br>• Small and pyknotic medulla neurons<br>Maternal co-exposure to parsley juice could partially or fully rescue phenotypes	
C57BL/6J	10 mg/L Cd in maternal drinking water during gestation and/or lactation	PND35-49, with gestational exposure<br>• Decreased time spent in target quadrant in MWM test<br>• Fewer visits to novel arm in the Y-maze test<br>• Decreased GABAaRa5 protein abundance (PND35)<br>• Increased GABAaRa5 protein abundance (PND49)	(Zhao et al., 2018)
Swiss albino mice	Maternal IP injection of 1.2 mg/kg Cd from GD14-21, until F_1 birth, with continued breeding to generate F_2, with or without quercetin supplementation	PND100<br>• Impaired memory in F_1 and F_2 mice that could be alleviated by maternal co-exposure with quercetin<br>• Increased activity and expression of oxidative stress enzymes (GSH S-transferase, catalase) in the F_1 brain	(Halder et al., 2019)
C57BL/6J and CAST/EiJ hybrid mice (both directions of cross)	1 or 50 ppm $CdCl_2$ in maternal drinking water for 5 weeks before and during gestation	PND0, 50 ppm<br>• Enlarged proportional brain masses<br>• Several molecular changes in the brain, (measured in females with C57BL/6J mothers only):<br>• Increased brain expression of genes related to myelination, oligodendrocytes, and RA<br>• Altered abundances of proteins related to cellular energy, hypoxia, myelin, and histone 1 subunits<br>• Increased abundance of 2-hydroxyvaleric acid, 2-hydroxycaproic acid, and xanthin in the brain<br>PND0, 1 and 50 ppm (measured in females with C57BL/6J mothers only):<br>• Increased abundance of brain RA<br>• Significantly reduced brain mitochondrial DNA content<br>6 months old, 50 ppm (C57BL/6J mothers only)<br>• Increased exploratory behavior<br>• Reduced social inhibition	(Hudson et al., 2021)
Zebrafish	Embryonic aqueous exposure to 100 µM Cd from 4-24 hpf	24 hpf<br>• Decreased head size<br>• Unclear boundaries between brain subdivisions<br>• Impaired commitment of neural progenitor cells<br>• Reduced expression of proneuronal genes<br>• Reduced differentiated neurons and glia in the facial sensory ganglia<br>• Reduced axonogenesis	(Chow et al., 2008)
Zebrafish	Embryonic aqueous exposure to 100 µM Cd from 4-24 hpf	24 hpf<br>• Impaired neuronal development in the retina that led to microphthalmia and blindness	(Chow et al., 2009)
Zebrafish	Embryonic aqueous exposure to 0, 1, 4, 16 nM Cd from 6-48 hpf	144 hpf, 4 and 16 nM<br>• Reduced swimming speed	(Zhang et al., 2012)
Zebrafish	Embryonic aqueous exposure to 0.1-10 µM Cd from 24-96 hpf	120 hpf<br>• Proportionally heavier brains	(Wold et al., 2017)

Disease Models & Mechanisms • Supplementary information

Zebrafish	Embryonic aqueous exposure to 100 or 200 μg/L Cd from 2-144 hpf, with or without Wnt activation<br><br>*behavior testing only conducted on 200 ug/L group	144 dpf, 100 and 200 ug/L Cd alone:<br>• Reduced β-catenin abundance<br>• Upregulation of Wnt inhibitors *ddk1* and *gsk3β*<br>• Downregulation of Wnt/catenin signaling targets: *lef1, axin2, myca, ccnd,* and *sp52*<br>144 dpf, 200 ug/L Cd alone:<br>• Delayed achievement of embryonic nervous system development milestones<br>• Shorter swimming distance and duration, and increased turning angle<br>With Wnt activation:<br>• Recovered Wnt signaling and motility and attenuation of developmental delays	(Xu et al., 2022b)
Zebrafish	Embryonic aqueous exposure to 2.5-200 μg/L Cd from 2-144 hpf	144 dpf, 50-200 μg/L:<br>• Dose responsive decrease in swimming distance, speed, acceleration, duration, and increase in turning angle<br>• Dose-responsive dysneurogenesis<br>• Reduced *a1-tubulin* and *nestin* transcript abundance<br>144 dpf, 100-200 μg/L:<br>• Reduced abundance of *neurogenin1* transcript<br>• Increased abundance of apoptosis related transcripts: *caspase-3a* and *caspase-9*<br>144 dpf, 200 μg/L:<br>• Reduced abundance of neurodevelopment-related transcripts: *shha, gfap, syn2a, gli2b, elavl3,* and *gap43*<br>• Increased neuronal abundance of apoptosis related transcripts: *bax* and *caspase-8*	(Xu et al., 2022a)
Zebrafish	Embryonic aqueous exposure to 40 ppb Cd from 4-120 hpf with or without Ca, Zn or NAC supplementation	120 hpf, Cd only:<br>• Increased rotational movement<br>• Hypersensitivity to auditory stimuli<br>• Reduced otolith size<br>120 hfp, Cd with Zn or NAC<br>• Cd induced phenotypes remained<br>120 hfp, Cd with Ca<br>• Cd-induced phenotypes recovered	(Green et al., 2023)
American bullfrog	Aqueous exposure to 1 μg/L $CdCl_2$ from 7-23 days old	7-23 days old<br>• Significantly reduced activity levels	(Dal-Medico et al., 2014)

Disease Models & Mechanisms • Supplementary information

Table S10. Key findings related to reproductive function from experimental animal models of early life Cd exposure

Species	Exposure Model	Key Findings	Source
Norway rat	Maternal SQ injection of 1 or 2 mg/kg Cd as $CdCl_2$ on GD6, with or without 1 or 2 mg/kg Se as sodium selenite (Na_2SeO_3)	PND21, 1 and 2 mg/kg Cd without Se:<br>• Cd accumulation in testes and ovaries<br>• Increase in diameter of seminiferous tubules<br>• Progressive sloughing of germ cells<br>• Vacuolization of Sertoli cells<br>• Hyperplasia of Leydig cells<br>• Reduction in the ovary size<br>PND21, 1 and 2 mg/kg Cd with Se:<br>• Recovery of Cd-induced histopathological changes	(Bekheet, 2011)
Wistar rat	Maternal oral exposure to 3 mg/kg Cd on GD15	GD 20, 3 mg/kg:<br>• No changes in expression of steroidogenesis genes encoding testicular steroidogenic acute regulatory protein (StAR) and pituitary luteinizing hormone (LH)	(Kariyazono et al., 2015)
	0.01 or 0.1 ppm $CdCl_2$ in maternal drinking water from GD1-20	GD 20, 0.01 and 0.1 ppm:<br>• Decreased transcript abundance of testicular StAR<br>• No significant change in transcript abundance of pituitary LH	
Sprague-Dawley rat	Paternal oral gavage exposure to 22.15 mg/kg $CdCl_2$ every 2 days for 9 weeks	PND21:<br>• Cd accumulation in testes<br>• Reduced SOD activity and GSH content in testes<br>• No change in MDA content in testes<br>PND70:<br>• Reduced SOD activity and GSH content in testes<br>• Increased MDA content in testes	(Zhao et al., 2015)
Charles Foster rat	Daily maternal SQ injection of 0.05 mg/kg CdAc from GD1-PND21	PND56:<br>• Reduced epididymis weight<br>• Reduced 17β-HSD and 3β-HSD activity in the testes<br>• Decreased serum T levels<br>• Diminished testicular and cauda-epididymal sperm counts<br>• Reduced cauda-epididymal sperm motility<br>• Derangements of the testes histoarchitecture, particularly in the testicular epithelium<br>• Decreased vitamin C and total cholesterol levels in testes<br>• Increased acid phosphatase activity in the testes<br>• Decreased vitamin C levels and acid phosphatase activity in the cauda-epididymitis<br>• Decreased fructose content in the seminal vesicle<br>• Diminished GSH and antioxidant (SOD, GPx, and CAT) levels in the testes and cauda-epididymis<br>• Increased TBARS levels in the testis and cauda epididymis	(Pillai et al., 2012)
Sprague-Dawley rat	Maternal IP injection of 5 or 50 µg/kg $CdCl_2$ on GD12 and GD17	PND0-38, 5 µg/kg:<br>• Earlier onset of vaginal opening<br>• Increased transcript abundance of gonadotropin releasing hormone (GnRH) in the hypothalamus<br>• Alterations in mammary gland development:<br>• Increased number of mammosphere-forming cells in neonatal mice	(Parodi et al., 2017)

Disease Models & Mechanisms • Supplementary information

		• Increased branching, number of epithelial cells, and density in prepubertal mice<br>• Increased mammary stem/progenitor cell populations, as well as overexpression and altered regulation of *ERα* in postpubertal mice<br>○ Increased expression of *Aldh1A1* in earlier prepubertal mice<br>PND0-38, 50 µg/kg:<br>• No change in onset of vaginal opening	
Wistar rat	50 mg/L Cd as $CdCl_2$ in maternal drinking water from during gestation and lactation, with or without 60 mg/L Zn as $ZnCl_2$	GD20, Cd alone<br>• No detected Cd or change in morphology in testes<br>Decreased concentration of Zn in testes<br>Gd20, Cd with Zn<br>• Recovery of testicular Zn<br>PND12, Cd alone<br>• Cd accumulation and reduced Zn in testes<br>• No change in testicular histology or seminiferous tubule diameters<br>PND12, Cd with Zn<br>• Partial recovery of testicular Zn<br>• Reduced Cd accumulation in testes<br>PND21, Cd alone<br>• No change in testicular histology or seminiferous tubule diameters<br>• Cd accumulation and reduced Zn in testes<br>PND21, Cd with Zn<br>• No recovery of testicular Zn<br>• Reduced Cd accumulation in testes<br>PND35, Cd alone<br>• Increased burden of Cd in testes<br>• Decreased relative testes weight<br>• Decreased levels of plasma T<br>• No change in testicular Zn, histology or seminiferous tubule diameters<br>• Increase in abnormal seminiferous tubules<br>PND35, Cd with Zn<br>• Recovery of plasma T<br>• Partial recovery of testicular Cd<br>• Recovery of testes weight<br>• Recovery of seminiferous tubules	(Chemek et al., 2016)
Sprague-Dawley rat	1, 5, 10 mg/L $CdCl_2$ in maternal drinking water from GD0-PND21	PND0-84, all groups<br>• No difference in the age at testes descent in males<br>• No difference in the age of vaginal opening in the females<br>• No changes in serum hormone levels (T, FSH, and LH) in males or females<br>• No differences in sperm counts or motility, or incidence of abnormal sperm were observed in male adults<br>• No change in the length of the estrous cycle of females<br>PND0-84, 10 ppm:<br>• Reduced testes weight	(Luo et al., 2015)
Wistar rat	10 mg/L CdAc in maternal drinking water from GD0-PND21	PND90:<br>• No change in ventral prostrate (VP) weight<br>• No difference in serum T levels<br>• No differences in cell proliferation and apoptosis indexes or androgen receptor immunostaining in VP<br>• Increased stromal inflammatory foci and multifocal inflammation of the VP	(Santana et al., 2016)

Wistar rat	10 mg/L CdAc in maternal drinking water from GD0-PND21	PND90:<br>• No change in the absolute and relative weights of male reproductive organs<br>• No changes in serum hormone levels (T, LH, and FSH)<br>• No differences observed in histopathological analyses of the testis and epididymis<br>• No changes in the number of mature spermatids in the testis, daily sperm production, the number of sperm in the caput/corpus and cauda-epididymis, or sperm transit time<br>• Decreases in sperm motility and morphology<br>• Increases in the rate of cell death in the testis	(Banzato et al., 2012)
Sprague-Dawley rat	Daily paternal oral gavage with 0.5, 2.0, and 8.0 mg/kg $CdCl_2$ from PND28-56	*F_1*<br>PND56-63, 0.5 mg/kg<br>• No changes in testicular morphology<br>• No change in rates or markers of apoptosis in GCs<br>• Decreased *Bcl2*, *Caspase8*, and *Caspase9* mRNA abundance but no changes in protein abundances in GCs<br>• Differential expression of apoptosis-related miRNAs in GCs<br>PND56-63, 2.0 mg/kg<br>• Incomplete cell nuclear membrane and abnormal sperm condensation in spermatogenic cells<br>• Increased lysosomes and decreased mitochondria but no apoptotic bodies in GCs<br>• Increased *Bax*, *Bcl-xl*, and *Caspase9* mRNA abundance but decreased *Bcl2* mRNA abundance in GCs<br>• Increased Bax, Bcl-xl, Cle-Caspase8, and Pro-Caspase9 protein abundance in GCs<br>• Differential expression of apoptosis-related miRNAs in GCs<br>PND56-63, 8.0 mg/kg<br>• Abnormal sperm morphology, impaired sperm condensation, enlarged intercellular space pyknotic nuclei, and ill-defined cell membranes in spermatogenic cells<br>• Increased rate of apoptosis, presence of apoptotic bodies, and organelle damage in GCs<br>• Increased *Bax*, *Bcl-xl*, *Caspase8*, and *Caspase9* mRNA abundance and Bax, Cle-Caspase3, Pro-caspase9, and Cle-Caspase9 protein abundance in GCs<br>• Differential expression of apoptosis-related miRNAs in GCs<br>• No change in the mean methylation levels of the Bax, Bcl2, Bcl-xl, Caspase3, Caspase8, and Caspase9 promoter regions in GCs<br>• Decreased methylation level at site 3 of the bcl-xl-1 fragment in GCs	(Sun et al., 2023b)

		F_2<br>PND56-63, 0.5 mg/kg<br>• No change in rates or markers of apoptosis in GCs<br>• Increased *Bcl2* and *Bcl-xl* mRNA abundance but no changes in protein abundances in GCs<br>• Differential expression of apoptosis-related miRNAs in GCs<br>PND56-63, 2.0 mg/kg<br>• Increased rate of apoptosis, presence of apoptotic bodies, and organelle damage in GCs<br>• Increased *Bcl-xl* and *Caspase9* mRNA expression and Bax, Cle-Caspase3, Pro-Caspase8, and Pro-Caspase9 protein abundance in GCs<br>• Differential expression of apoptosis-related miRNAs in GCs<br>PND56-63, 8.0 mg/kg<br>• Increased rate of apoptosis, presence of apoptotic bodies, and organelle damage in GCs<br>• Increased *Bax, Bcl-xl, Caspase3*, *Caspase8*, and *Caspase9* mRNA abundance and Cle-Caspase3 and Cle-Caspase9 protein abundance in GCs<br>• Differential expression of apoptosis-related miRNAs in GCs<br>• No change in the mean methylation levels of the Bax, Bcl2, Bcl-xl, Caspase3, Caspase8, and Caspase9 promoter regions in GCs<br>• Increased methylation level at site 21.22 of the Bax fragment and site 11.12 of the caspase 9 fragment in GCs	
Sprague-Dawley rat	Daily maternal (F_0) oral gavage with 0.5, 2.0, and 8.0 mg/kg $CdCl_2$ from GD0-PND0	PND70, Cd alone<br>• Decreased relative testes weight, relative seminal vesicle weight, and relative epididymis weight<br>• Altered testicular structure and increased percentage of abnormal seminiferous tubules characterized by germ cells loss, intercellular vacuolization, desquamation of spermatogenic cells from the basement membrane, and cell exfoliation in the lumen<br>• Decreased Daam1 protein and *Daam1* mRNA abundance in testes<br>• Decreased daily sperm production (DSP) and efficiency of sperm production in testes<br>• Decreased epididymal sperm concentration and mobility<br>• Increased abnormalities in the head and tail of sperm in the epididymis<br>PND70, Cd and Zn<br>• Recovery of relative testes, seminal vesicle, and epididymis weights<br>• Amelioration of Cd-induced testicular damage characterized by restored histological structure of the testicular tissue<br>• Recovery of Daam1 protein and *Daam1* mRNA abundance in testes<br>• Amelioration of Cd-induced decrease in daily sperm production (DSP) and efficiency of sperm production in testes<br>• Recovery of epididymal sperm concentration and mobility	(Liu et al., 2021b)

Disease Models & Mechanisms • Supplementary information

		• Partial amelioration of the increased abnormalities in the head and tail of sperm in the epididymis	
		F_2<br>PND56, 8.0 mg/kg:<br>• Swollen mitochondria in OGCs<br>• No change in apoptosis rate in OGCs<br>• Increase in *Bcl2*/Bcl2 transcript and protein abundance in OGCs<br>• Altered expression of miRNAs that regulate Cd-induced apoptosis in OGCs	
		F_3<br>PND56, 8.0 mg/kg:<br>• No change in *Bcl2* gene expression in OGCs<br>• Altered expression of miRNAs that regulate Cd-induced apoptosis in OGCs	
Sprague-Dawley rat	Daily maternal (F_0) oral gavage with 0.5, 2.0, and 8.0 mg/kg $CdCl_2$ from GD0-PND0	*F_1*<br>PND56, 0.5 mg/kg:<br>• No change in levels but decreased P4 levels in OGCs<br>• Alterations in steroidogenic enzymes in OGCs:<br>○ Increased sf-1 transcript abundance but no change in protein abundance<br>○ Decreased StAR mRNA and protein abundance<br>○ Decreased Cyp11a1 mRNA and protein abundance<br>PND56, 2.0 mg/kg:<br>• No change in E2 levels but decreased P4 levels in OGCs<br>• Alterations in steroidogenic enzymes in OGCs:<br>○ Decreased sf-1 transcript abundance but no change in protein abundance<br>○ Decreased StAR mRNA and protein abundance<br>○ Decreased Cyp11a1 mRNA and protein abundance<br>PND56, 8.0 mg/kg<br>• No change in E2 levels but decreased P4 levels in OGCs<br>• Alterations in steroidogenic enzymes in OGCs:<br>○ Increased sf-1 transcript abundance but no change in protein abundance<br>○ Decreased StAR mRNA and protein abundance<br>○ Decreased Cyp11a1 mRNA and protein abundance<br>• Altered expression of miRNAs that regulate Cd-induced steroidogenesis in OGCs	(Liu et al., 2020)
		F_2<br>PND56, 8.0 mg/kg<br>• No change in E2 levels but decreased P4 levels in OGCs<br>• Alterations in steroidogenic enzymes in OGCs:<br>○ Decreased sf-1 mRNA and protein abundance<br>○ Increased StAR transcript abundance but decreased protein abundance<br>○ Increased Cyp11a1 transcript abundance but decreased protein abundance	

Disease Models & Mechanisms • Supplementary information

		Altered expression of miRNAs that regulate Cd-induced steroidogenesis in OGCs	
Sprague-Dawley rat	Daily maternal (F_0) oral gavage with 0.5, 2.0, and 8.0 mg/kg $CdCl_2$ from GD0 to PND0	*F_1*<br>PND21, 0.5 mg/kg:<br>• Increased seminiferous tubules (ST) diameter in testes<br>• Reduced T<br>• No difference in expression of steroidogenic genes<br>• Reduced protein abundance steroidogenic enzyme CYP11A1<br>PND21, 2.0 mg/kg:<br>• Increased ST diameter in testes<br>• Reduced T<br>• Downregulation of steroidogenic *Star* gene in testes<br>• Reduced protein abundance of Star and Cyp11a1 in testes<br>PND21, 8.0 mg/kg:<br>• Increased ST diameter in testes<br>• Increased chromatin aggregation in the spermatogonial nuclei and cell fragmentation in testes<br>• Decreased serum GnRH, P4, and T levels<br>• Reduced gene expression of *sf-1* and other steroidogenic enzymes in testes, but not *Star*<br>• Decreased protein abundance of SF-1 and STAR in testes<br>PND56, 0.5 mg/kg:<br>• Decreased ST diameter and decreased spermatogenesis activity in testes<br>• Reduced HSD3β and CYP17A1 in testes<br>PND56, 2.0 mg/kg:<br>• No change in ST diameter or spermatogenesis activity in testes<br>• Decreased serum LH levels<br>• Increased expression of *sf-1* in testes<br>• Reduced SF-1 protein in testes<br>PND56, 8.0 mg/kg:<br>• Increased injury of tight junctions between spermatogenic cells, vacuolization within the cells, and shrunken spermatogonia and nuclei<br>• No change in serum P4 but decreased GnRH, LH, and T levels<br>• No change in *Star* gene expression but increased expression of *Sf-1* and other steroidogenic enzymes in testes<br>• No change in SF-1, STAR, or other steroidogenic enzymes protein levels in testes besides decreased CYP11A1	(Huang et al., 2020)

		F_2<br>PND21, 8.0 mg/kg:<br>• Decreased ST diameter in testes<br>• Increased nucleolar chromatin aggregation, nucleolar concentration and cytoplasm dehydration with vacuoles in testes<br>• No change in serum GnRH, LH, or P4 but increased T levels<br>• Increased *sf-1* and *star* gene expression in testes<br>• Reduced STAR and HSD3β protein in testes<br>PND56, 8.0 mg/kg:<br>• Increased ST diameter and decreased spermatogenesis activity in testes<br>• Increased cell membranolysis and basement membrane thickness variation in testes<br>• No change in serum GnRH, LH, or P4 but increased T levels<br>• Increased STAR and other steroidogenic enzymes protein levels in testes	
Sprague-Dawley rat	Daily maternal oral gavage of 1 mg/kg or 5mg/kg $CdCl_2$ from GD0 to PND21	*F_1*<br>PND21-45, 1 mg/kg:<br>• No change<br>PND21-45, 5 mg/kg:<br>• Decreased age of vaginal opening and first day of estrus<br>• Increased F_2 litter size<br>PND21, 1 mg/kg:<br>• No Cd accumulation in reproductive organs<br>• Decreased number of primordial follicles in ovaries<br>• Increased hypertrophy and epithelial hyperplasia in uterus<br>• No change in serum P4 levels but increased E2<br>• Marginal activation of DLD-cAMP/PKA-ERK1/2 signaling pathway in ovaries<br>• Up-regulation of steroidogenic enzyme CYP11A1 in ovaries<br>PND35, 1 mg/kg:<br>• No Cd accumulation in reproductive organs<br>• No change in relative ovary weight but increased relative uterus weight<br>• Hypertrophy and epithelial hyperplasia in uterus<br>• No change in serum P4 levels but increased E2<br>• No activation of DLD-cAMP/PKA-ERK1/2 signaling pathway in ovaries<br>• Up-regulation of steroidogenic enzyme StAR in ovaries<br>PND56, 1 mg/kg:<br>• No change in Cd concentrations in ovaries or uterus<br>• No change in relative ovary weight but increased relative uterus weight<br>• No change in follicle numbers in ovaries<br>• Increased hypertrophy and epithelial hyperplasia in uterus<br>• No change in serum P4 or E2 levels<br>• Activation of DLD-cAMP/PKA-ERK1/2 signaling pathway in ovaries<br>• Up-regulation of steroidogenic enzyme StAR in ovaries<br>PND21, 5 mg/kg:<br>• Increased Cd burden in ovaries and uterus<br>• No change in relative ovary weight but increased relative uterus weight	(Li et al., 2018b)

Disease Models & Mechanisms • Supplementary information

		• Decreased number of primordial follicles but increased secondary and antral follicles in ovaries<br>• Increased hypertrophy and epithelial hyperplasia in uterus (more severe than 1 mg/kg group)<br>• Increased serum P4 and E2 levels<br>• Activation of DLD-cAMP/PKA-ERK1/2 signaling pathway in ovaries<br>• Up-regulation of steroidogenic enzyme CYP19A1 in ovaries<br>PND35, 5 mg/kg:<br>• Increased Cd burden in ovaries and uterus<br>• No change in relative ovary weight but increased relative uterus weight<br>• Decreased number of primordial follicles but increased secondary and antral follicles in ovaries<br>• Increased hypertrophy and epithelial hyperplasia in uterus (more severe than 1 mg/kg group)<br>• Increased serum P4 and E2 levels<br>• Activation of DLD-cAMP/PKA-ERK1/2 signaling pathway in ovaries<br>• Up-regulation of steroidogenic enzymes StAR, CYP11A1, and CYP19A1 in ovaries<br>PND56, 5 mg/kg:<br>• No change in Cd concentrations in uterus but increased burden in ovaries<br>• Increased relative ovary and uterus weight<br>• Decreased number of primordial follicles but increased secondary and antral follicles in ovaries<br>• Increased hypertrophy and epithelial hyperplasia in uterus (more severe than 1 mg/kg group)<br>• Activation of DLD-cAMP/PKA-ERK1/2 signaling pathway in ovaries<br>• Up-regulation of steroidogenic enzymes StAR, 3β-HSD, CYP11A1 and CYP19A1 in ovaries	
		F_2<br>PND21, 5 mg/kg:<br>• Decreased number of primordial follicles but increased antral follicles in ovaries<br>• Increased serum P4 and E2 levels<br>• No change in serum E2 levels but increased P4<br>• Up-regulation of CYP11A1 and CYP19A1 in ovaries<br>PND35, 5 mg/kg:<br>• Up-regulation of CYP11A1 in ovaries<br>PND56, 5 mg/kg<br>• Up-regulation of CYP19A1 in varies	
Sprague-Dawley rat	Daily maternal oral gavage of 1 or 5 mg/kg $CdCl_2$ from GD0-PND21	PND21, 1 and 5 mg/kg:<br>• Decreased relative weight of testes<br>• Reduced phosphorylation of P-ERK1/2 in testes<br>• Decreased abundance of steroidogenic enzymes CYP11A1 and 3β-HSD<br>PND21 5 mg/kg only:<br>• Cd accumulation in testes<br>• Increased serum P4<br>• Reduced serum androstenedione<br>• Reduced protein abundance of PKA in testes<br>• Reduced abundance of steroidogenic enzymes StAR and CYP17A1<br>PND35, 1 and 5 mg/kg:	(Tian et al., 2018)

Disease Models & Mechanisms • Supplementary information

		• Decreased serum P4<br>• Decreased abundance of steroidogenic enzyme 3β-HSD<br>PND35, 5 mg/kg only:<br>• Cd accumulation in testes<br>• Reduced abundance of steroidogenic enzyme CYP11A1<br>• Reduced protein abundance of PKA and phosphorylation of ERK1/2<br>PND56, 1 and 5 mg/kg:<br>• Reduced abundance of steroidogenic enzyme CYP11A1<br>PND56, 5 mg/kg only:<br>• Decreased relative weight of testes<br>• Decreased serum T<br>• Reduced protein abundance of PKA and phosphorylation of ERK1/2	
Sprague-Dawley rat	Maternal IP injection of 0.25, 0.5 or 1.0 mg/kg Cd on GD12	GD20, 0.25 mg/kg:<br>• No formation of multinucleated gonocytes in testes<br>• No change in testicular T levels<br>• No change in Leydig cell numbers in testes<br>• Decreased mRNA and protein abundance of Leydig cell-specific and Sertoli cell-specific genes in testes<br>• Decreased Leydig cell and cytoplasm size<br>GD20, 0.5 mg/kg:<br>• No formation of multinucleated gonocytes in testes<br>• Decreased testicular T levels<br>• Decreased Leydig cell numbers in testes<br>• Decreased mRNA and protein abundance of Leydig cell-specific and Sertoli cell-specific genes in testes<br>• Decreased Leydig cell and cytoplasm size<br>GD20, 1.0 mg/kg:<br>• No formation of multinucleated gonocytes in testes<br>• Decreased testicular T levels<br>• Decreased Leydig cell numbers in testes<br>• Decreased mRNA and protein abundance of Leydig cell-specific and Sertoli cell-specific genes in testes<br>• Decreased Leydig cell and cytoplasm size	(Li et al., 2018a)
Wistar rat	50 and 200 ppm $CdCl_2$ in maternal drinking water from GD9-21	PND22-60, 50 ppm:<br>• Delayed onset of vaginal opening<br>• Altered estrous cyclicity<br>PND22-60, 200 ppm:<br>• Delayed onset of vaginal opening<br>• Altered estrous cyclicity<br>PND10, 50 ppm:<br>• Altered primary and secondary follicles in ovaries<br>• Decreased antioxidant enzymes protein levels in ovaries<br>• Increased lipid peroxidation enzymes protein levels in ovaries<br>• No change in serum P4 levels but decreased T and E2<br>PND21, 50 ppm:<br>• Severe disorganization and damage of ovarian histoarchitecture with increased numbers of follicles at various stages of degeneration and scattered graffian follicles throughout the ovary without oocytes	(Samuel et al., 2011)

Disease Models & Mechanisms • Supplementary information

		• Decreased antioxidant enzymes protein levels in ovaries<br>• Increased lipid peroxidation enzymes protein levels in ovaries<br>• No change in serum P4 levels but decreases T and E2<br>PND10, 200 ppm:<br>• Decreased ovary weight<br>• Disorganized secondary follicles with altered oocytes in ovaries<br>• Decreased antioxidant enzymes protein levels in ovaries<br>• Increased lipid peroxidation enzymes protein levels in ovaries<br>• Decreased serum T, E2, and P4 levels<br>PND21, 200 ppm:<br>• Decreased ovary weight<br>• Severe disorganization and damage of ovarian histoarchitecture with increased numbers of follicles at various stages of degeneration, remnants of disorganized granulosa cells, and cumulus oophorus dysfunction<br>• Decreased antioxidant enzymes protein levels in ovaries<br>• Increased lipid peroxidation enzymes protein levels in ovaries<br>• Decreased serum T, E2, and P4 levels	
Wistar rat	10 mg/L Cd from CdAc in maternal drinking water from GD0-PND5	PND0:<br>• No change in testes or ovary weight<br>• Decreased seminiferous tubule diameter (STD) and prospermatogonia (Pro-SPG) numbers in testes<br>• Decreased RNA and DNA contents in testes<br>• Decreased DNA content in ovaries<br>PND5:<br>• Decreased testes and ovary weight<br>• Decreased STD and Pro-SPG numbers in testes<br>• Decreased DNA content in testes<br>• Decreased DNA content in ovaries	(Corpas and Antonio, 1998)
Wistar rat	Daily maternal oral gavage of 20 mg/kg Cd from $CdCl_2$ from GD6-14	PND1-40:<br>• Delay in age of vaginal opening<br>• Earlier descent of testes<br>PND180:<br>• Decreased mount latency, increased number of mounts, and increased number of intromissions in males<br>• Decreased Lordosis coefficient (measure of sexual receptivity) in females	(Salvatori et al., 2004)

Disease Models & Mechanisms • Supplementary information

Sprague-Dawley rat	Daily maternal intragastric exposure to 0.5, 1.0, and 2.0 mg/kg of $CdCl_2$ from GD0-PND0	*F_1*<br>PND5, All doses<br>• Decreased diameter of seminiferous tubules (STs) in the 1.0 mg/kg group<br>• Increased necrosis, vacuolization, reduction of nuclear density, and impaired spermatogenic cell connection in Sertoli cells in the 2.0 mg/kg group<br>• No change in serum GnRH levels but increased FSH levels in the 2.0 mg/kg group<br>• Increased *Fshr* mRNA abundance at 0.5 mg/kg, increased Akt protein abundance at 1 mg/kg, and increased Foxo1 protein abundance at 2.0 mg/kg<br>• Decreased *Dnmt3a* mRNA abundance at 1.0 mg/kg but increased *Dnmt3b* mRNA abundance at 2.0 mg/kg<br>• Increased Dnmt3a and Dnmt3b protein abundance at 2.0 mg/kg<br>PND21, All doses<br>• No change in STs diameter<br>• Increased serum GnRH levels in the 0.5 mg/kg group but decreased FSH levels in all dosing groups<br>• Increased *Foxo1* mRNA abundance but decreased Foxo1 protein abundance at 0.5 mg/kg<br>• Increased Fshr and Akt protein abundance at 2.0 mg/kg<br>• No change in the general DNA methylation of *Fshr*, *Akt* and *Foxo1* genes in the 2.0 mg/kg group<br>• Decreased methylation levels at position 65 sites of the *Foxo1-14* fragment and position 28 and 29 sites of the *Foxo1-17* fragment in the 2.0 mg/kg group<br>• Increased *Dnmt1* and *Dnmt3a* mRNA abundance at 0.5 mg/kg but no changes in protein abundances in any groups<br>PND56, All doses<br>• No change in STs diameter<br>• No change in the Johnsen score (a scoring system used to assess spermatogenesis) in testes<br>• Increased lysis, vacuolization, atrophy and necrosis, reduction in matrix density, and impaired connection with surrounding cells in Sertoli cells in the 2.0 mg/kg group<br>• No change in serum GnRH or FSH levels<br>• Decreased area of density (AOD) of Fshr protein after immunohistochemistry staining of Sertoli cells in the 2.0 mg/kg group<br>• Increased *Akt* mRNA abundance and Foxo1 protein abundance at 1.0 mg/kg<br>• No change in the general DNA methylation of *Fshr*, *Akt* and *Foxo1* genes in the 2.0 mg/kg group<br>• Decreased methylation level of *Akt-9* fragment at the second position site in the 2.0 mg/kg group<br>• Increased *Dnmt3a* mRNA abundance at 2.0 mg/kg and Dnmt3b protein abundance at 1.0 and 2.0 mg/kg	(Li et al., 2023)

Disease Models & Mechanisms • Supplementary information

		F₂<br>PND5, All doses<br>• No change in STs diameter<br>• Increased serum FSH levels in 0.5 mg/kg group but no change in GnRH levels<br>• Increased *Pi3k*, *Akt*, and *Foxo1* mRNA abundance at 2.0 mg/kg but no changes in protein abundances at any dose<br>• No changes in mRNA abundances at any of the doses<br>• Increased Dnmt1 protein abundance at 2.0 mg/kg<br>PND21, All doses<br>• No change in STs diameter<br>• No change in serum GnRH or FSH levels<br>• Decreased *Fshr* mRNA abundance at 0.5 and 1.0 mg/kg<br>• Increased PI3k protein abundance at 2.0 mg/kg, decreased Akt protein abundance at 1.0 mg/kg, and increased Foxo1 protein abundance at 1.0 mg/kg<br>• No change in the general DNA methylation of *Fshr*, *Akt* and *Foxo1* genes in the 2.0 mg/kg group<br>• Decreased methylation level at the fourth position site of the *Fshr-8* fragment, decreased methylation level at the 12th position site of the *Akt-33* fragment, and increased methylation level at the 21st and 22nd sites of the *Foxo1-15* fragment in the 2.0 mg/kg group<br>• Decreased *Dnmt3a* mRNA abundance at 1.0 mg/kg but no changes in protein abundances at any of the doses<br>PND56, All doses<br>• Decreased diameter of STs in 0.5 mg/kg group but increased diameter of STs in 2.0 mg/kg group<br>• No change in Johnsen score in testes<br>• No change in serum GnRH or FSH levels<br>• No change in the AOD of Fshr protein after immunohistochemistry staining of Sertoli cells in the 2.0 mg/kg group<br>• No changes in mRNA abundances at any dose<br>• Increased Akt protein abundance at 1.0 and 2.0 mg/kg and Foxo1 protein abundance at 2.0 mg/kg<br>• No change in the general DNA methylation of *Fshr*, *Akt* and *Foxo1* genes in the 2.0 mg/kg group<br>• Decreased *Dnmt3a* mRNA abundance at 0.5 and 2.0 mg/kg but no changes in protein abundance at any of the doses	
Sprague-Dawley rat	Daily paternal oral gavage with 0.5, 2.0, and 8.0 mg/kg $CdCl_2$ from PND28-56	*F₁*<br>PND40-55, All doses<br>• No change in the ovarian organ coefficient or vaginal opening time in females<br>• Decreased duration of diestrus and total estrous cycle in the 8 mg/kg group females<br>Adult, 0.5 mg/kg<br>• No change in serum Pg and E2 levels in females<br>• Decreased *Star*, *Sf-1*, and *Cyp11a1* mRNA abundance but increased Cyp11a1 and Sf-1 protein abundance in GCs	(Sun et al., 2023a)

Disease Models & Mechanisms • Supplementary information

		• Changes in hormone synthesis-related miRNAs in GCs<br>• Increased *Igf2* mRNA abundance in GCs<br>Adult, 2.0 mg/kg<br>• Decreased serum Pg and E2 levels in females<br>• Decreased *Cyp11a1* mRNA abundance and Star protein abundance but increased *Cyp19a1* mRNA abundance in GCs<br>• Changes in hormone synthesis-related miRNAs in GCs<br>• Increased *H19 and Peg3* mRNA abundance in GCs<br>Adult, 8.0 mg/kg<br>• Decreased serum Pg and E2 levels in females<br>• Decreased *Star* and *Cyp11a1* mRNA abundance and Star protein abundance but increased *Cyp19a1* mRNA abundance in GCs<br>• No change in the mean methylation levels of Cyp11a1, Cyp19a1, Sf-1, and star in GCs<br>• Increased methylation levels at site 13 of the Cyp11a1-14 fragment, site 7.8.9.10.11 of the Sf-1 fragment, and site 5 of the Star-18 in GCs<br>• Changes in hormone synthesis-related miRNAs in GCs<br>• Increased *Igf2*, *Kcnq1*, *Mest*, *Peg3*, *Peg12*, and *Snrpn* mRNA expression in GCs<br>Increased *Igf2*, *Peg12*, *Mest*, and *Snrpn* mRNA abundance but decreased *H19*, *Peg3*, and *Kcnq1* mRNA abundance in sperm	
		F_2<br>PND40-55, All doses<br>• No change in the ovarian organ coefficient in females<br>• Increased ovarian organ coefficient in the 2 mg/kg group females<br>• Increased durations of metestrus, diestrus, and total estrous cycle in the 8 mg/kg group females<br>• Increased duration of estrous in the 2 mg/kg group females<br>• Decreased proestrus duration in the 0.5 mg/kg group females<br>Adult, 0.5 mg/kg<br>• No change in serum E2 levels but increased E2 levels in females<br>• Increased *Star*, *Cyp11a1*, and *Cyp19a1* mRNA abundance and Cyp19a1, Star, and Sf-1 protein abundance in GCs<br>• Changes in hormone synthesis-related miRNAs in GCs<br>• No changes in imprinted gene mRNA abundance in GCs<br>Adult, 2.0 mg/kg<br>• No change in serum E2 levels but increased E2 levels in females<br>• Increased *Star*, *Sf*-1, *Cyp11a1*, and *Cyp19a1* mRNA abundance and Cyp19a1, Star, and Sf-1 protein abundance in GCs<br>• Changes in hormone synthesis-related miRNAs in GCs<br>• Increased *H19*, *Igf2*, *Kcnq1*, and *Snrpn* mRNA abundance in GCs<br>Adult, 8.0 mg/kg<br>• No change in serum E2 levels but increased E2 levels in females	

Disease Models & Mechanisms • Supplementary information

		• Increased *Star*, *Sf*-1, *Cyp11a1*, and *Cyp19a1* mRNA abundance and Cyp19a1 and Star protein abundance in GCs<br>• No changes in the mean methylation levels of Cyp11a1, Cyp19a1, Sf-1, or Star or in the methylation levels at each site of the gene fragment in GCs<br>• Changes in hormone synthesis-related miRNAs in GCs<br>Increased *H19, Igf2*, *Kcnq1*, *Mest*, *Peg3*, *Peg12*, and *Snrpn* mRNA expression in GCs	
Sprague-Dawley rat	Maternal IP injection of 1.5 mg/kg $CdCl_2$ on GD9, 11, 13 and every other day during lactation, with or without 50 mg/kg tamoxifen (TMX)	GD18, Cd alone:<br>• Disordered arrangement of Sertoli cells and gonocytes in seminiferous tubules<br>GD18, Cd with TMX<br>• Recovery of seminiferous tubule disorder<br>PND30: Cd alone<br>• Decreased testes weight<br>• Increased ultrastructural damage of testes:<br>○ Swollen mitochondria in Leydig cells<br>○ Formation of myeloid structure<br>○ Seminiferous tubule degeneration<br>• Decreased expression of StAR protein and Inhibin-B mRNA in Leydig cells<br>PND30, Cd with TMX<br>• Recovery of testes weight<br>• Recovery of ultrastructural testes damage<br>• Partial recovery of StAR protein abundance<br>• Recovery of inhibin-B gene expression	(Liu et al., 2021a)
Sprague-Dawley rat	Daily maternal oral gavage with 0.5, 2.0, and 8.0 mg/kg $CdCl_2$ from GD1-20	*F_2*<br>PND56-60, 0.5 mg/kg<br>• No change in serum Pg levels but decreased serum E2 levels<br>• Increased *Cyp11a1* mRNA abundance and Cyp19a1 protein abundance but decreased Star and Cyp11a1 protein abundance in ovarian granulosa cells (GCs)<br>PND56-60, 2.0 mg/kg<br>• Decreased serum Pg and E2 levels<br>• Increased *Sf-1*, *Star*, and *cyp11a1* mRNA abundance but decreased *Cyp19a1* mRNA abundance and Sf-1, Star, and Cyp11a1 protein abundance in GCs<br>PND56-60, 8.0 mg/kg<br>• No change in serum Pg levels but decreased serum E2 levels<br>• Increased *Cyp11a1* and *Cyp19a1* mRNA abundance and Sf-1 protein abundance in GCs<br>• 35 differentially expressed miRNAs in GCs<br>○ Identified miRNA target genes were related to growth- and development-related gene ontology (GO) terms<br>• 50 and 61 gene promoter regions were hypermethylated and hypomethylated, respectively in GCs<br>GO analysis of hormone synthesis-related miRNA target genes and GO analysis of genes with differential DNA methylation revealed enrichment in the cAMP/PKA signaling	(Luo et al., 2023)

Disease Models & Mechanisms • Supplementary information

		F_3<br>PND56-60, 0.5 mg/kg<br>• Increased serum Pg levels but no change in serum E2 levels<br>• Increased *Cyp19a1* mRNA abundance and Sf-1 protein abundance but decreased Cyp11a1 protein abundance in GCs<br>PND56-60, 2.0 mg/kg<br>• Increased serum Pg and E2 levels<br>• Increased *Star*, *Cyp11a1*, and *Cyp19a1* mRNA abundance and Sf-1 and Cyp19a1 protein abundance but decreased Cyp11a1 protein abundance in GCs<br>PND56-60, 8.0 mg/kg<br>• Increased serum Pg and E2 levels<br>• Increased *Sf-1*, *Star*, *Cyp11a1*, and *Cyp19a1* mRNA abundance and Sf-1 and Cyp19a1 protein abundance but decreased Star and Cyp11a1 protein abundance in GCs<br>• 16 differentially expressed miRNAs in GCs<br>○ Identified miRNA target genes were related to growth- and development-related GO terms<br>• 54 1,136 gene promoter regions were hypermethylated and hypomethylated, respectively in GCs<br>GO analysis of hormone synthesis-related miRNA target genes and GO analysis of genes with differential DNA methylation revealed enrichment in the cAMP/PKA signaling	
ICR mouse	Maternal IP injection of 5-6 mg/kg $CdCl_2$ on GD 7.5 or 8.5	GD13.5:<br>• Reduced genital ridges in males and females<br>• Fewer primordial germ cells in males<br>• A large number of primordial germ cells were found outside the genital ridges in males and females<br>GD16.5:<br>• Reduced ovary and testes size<br>• Reduced number of differentiating germ cells in males and females<br>PND56-63:<br>• Increased incidence of sterility in males<br>○ Decreased litter sizes<br>○ Reduced testes size<br>○ Lowered fertilizing capability of spermatozoa<br>• No effect on fertility in females	(Tam and Liu, 1985)
CD-1 mouse	Maternal IP injection of 0.5 mg/kg $CdCl_2$ from GD13-17	GD18:<br>• Reduced absolute and relative testes weights of the male fetuses<br>• Decreased testicular mRNA and protein levels of StAR and T biosynthesis enzymes (P450scc, $P450_{17\alpha}$, and 17β-HSD)<br>PND70:<br>• Decreased testicular StAR mRNA and P450scc mRNA and protein levels, but no changes in $P450_{17\alpha}$ and 17β-HSD mRNA or protein levels<br>• No abnormal morphological features or increases in apoptotic cells in testes<br>• Reduced serum and testicular T<br>• No differences in the numbers of spermatozoa, weights of the testes or epididymides, or testes histology<br>• No effect on male fertility or ability to mate<br>• Increased resorptions per litter and decreased live fetuses per litter in F2 generation	(Ji et al., 2011)

C57BL/6J mouse	10 mg/L $CdCl_2$ in maternal drinking water from GD0-PND21	PND21:<br>• No change in serum T levels in males<br>PND35:<br>• No change in serum E2 levels in females<br>• No change in serum T levels in males<br>PND49:<br>• Reduced serum E2 levels in females<br>• No change in serum T levels in males<br>PND84:<br>• No change in serum E2 levels in females<br>• No change in serum T levels in males	(Zhao et al., 2018)
C57BL/6J mouse	1, 10, 100 µg/L $CdCl_2$ in maternal and paternal drinking water for 1, 3, or 5 months before mating with maternal exposure continuing from GD0- PND21 and offspring exposure continuing via drinking water from PND21 until PND70 (receiving same dose as parents)	PND70, all groups<br>• No difference in gonadal histopathology<br>• No difference in serum hormone levels<br>• No difference in TUNEL-positive cells in the testes or ovaries<br>• No difference in gonadal MDA and SOD levels<br>• No difference in testicular seminiferous tubule diameter or Leydig cell number<br>• No difference in sperm count, motility or morphology<br>PND70, F1 5-month exposure to 100 µg/L<br>• Changes in spermatogenic epithelial staging (stages VII and VIII) in the testes of males<br>• Decreased expression level of *Cyp17a1* (a Leydig cell specific gene) in the testes of males	(Zhang et al., 2019)
C57BL/6J Jcl mouse	1 or 10 ppm $CdCl_2$ in maternal drinking water from GD0-PND10	PND21-70, 1 ppm:<br>• No change in age at vaginal opening or normal cyclicity of estrous<br>PND21-70, 10 ppm:<br>• No change in age at vaginal opening or normal cyclicity of estrous	(Ishitobi and Watanabe, 2005)
C57BL/6J mouse	Daily paternal IP injection with 1.0 mg/kg $CdCl_2$ for 5 weeks	PND6<br>• Increased atrophy of seminiferous tubules, number of lipid droplets in testicular interstitial, and damaged and vacuolated mitochondria in the testes<br>• Transcriptomic analyses of testicular tissues revealed differentially expressed genes enriched in categories related to fatty acid synthesis and catabolism, flagellar movement of sperm and ion channels<br>• Increased *Fasn* and *Acat1* mRNA abundance but decreased *Ppard*, *Acsm5*, *Scd1*, *Pck1*, *Cd36*, and *Cyp7a1* mRNA abundance in the testes<br>PND70<br>• Increased testicular index and epididymal fat pad index in testes<br>• Decreased serum T levels in males<br>• Increased total serum cholesterol levels but decreased serum free cholesterol levels in males<br>• Decreased sperm concentration in the cauda epididymis<br>• Decreased *Star*, *P450scc*, *3β-hsd*, and *17β-hsd* mRNA and protein abundance in testes<br>• Increased lipid droplets in the testicular interstitial of the testes<br>• Decreased *Atgl, Hsl, Ldlr* and *Sr-bi* mRNA abundance and Atgl, Sr-Bi, and Ldlr protein abundance in testes<br>Decreased immunofluorescence and protein abundance of mitochondrial and lysosome markers Vdac1/2 and Lamp2 in the testes	(Zhou et al., 2022)

C57BL/6J mouse	Paternal IP injection with 1.0 mg/kg $CdCl_2$ every other day for 5 weeks	PND6<br>• Increased mitochondria in Sertoli cells, type A spermatogonia, and interstitial cells of testes<br>• Increased immunofluorescence and protein abundance of mitochondrial and lysosome markers Vdac1/2 and Lamp2 in the testes<br>PND112<br>Decreased immunofluorescence and protein abundance of mitochondrial and lysosome markers Vdac1/2 and Lamp2 in the testes	(Zeng et al., 2022)
C57BL/6J mouse	Paternal IP injection with 1.0 mg/kg $CdCl_2$ every other day for 5 weeks	PND6<br>• Increased number of mitochondria in oocytes with increased vacuolization and broken outer membranes<br>• Increased mitochondrial vacuolization but decreased mitochondrial cristae in GCs<br>PND70<br>• Increased ovary index<br>• Decreased serum E2 levels but no change in serum AMH levels<br>• Decreased number of secondary follicles and antral follicles in the ovaries<br>• Decreased *Star, P450scc* and *17β-hsd* mRNA abundance in ovaries<br>• Decreased immunofluorescence of Star and 17β-hsd in ovaries<br>• Decreased P450scc, 17β-hsd, Cyp17a1 and Cyp19a1 protein abundance in ovaries<br>• Increased total serum cholesterol levels but decreased serum free cholesterol levels in females<br>• Decreased total cholesterol in ovaries<br>• Decreased *Hmgcr, Srebp2, Ldlr* and *Abca1* mRNA abundance in ovaries<br>• Decreased Hmgcr, Srbi, and Abca1 protein abundance but no change in Ldlr in ovaries<br>• Increased lipid deposition in ovaries<br>• Decreased<br>• Decreased *Hsl* mRNA abundance but no chance in *Atgl* in ovaries<br>• Decreased immunofluorescence and protein abundance of Atgl and Hsl in ovaries<br>• Decreased *Drp1* and *Mfn2* mRNA abundance in ovaries<br>• Decreased immunofluorescence and protein abundance of Drp1 and Mfn2 in ovaries<br>No change in Opa1 and Mfn1 protein abundance in ovaries	(Zeng et al., 2023)
CD-1 mouse	100 mg/L $CdCl_2$ in paternal drinking water for 20 weeks	PND26-35:<br>• No change in anogenital distance, testicular descent, or weight of testes<br>PND70:<br>• No change in epididymal fat weight, prostate weight, or epididymal weight<br>• No change in staging of seminiferous tubules, histological morphology, or the number of atypical residual bodies per seminiferous tubule<br>• No increase in apoptosis of seminiferous tubules	(Nan et al., 2020)
NMRI mouse	Primary mouse testis and ovary tissue cultures exposed *ex vivo* to 1 or 10 µM Cd (from $CdCl_2$) for 3 days	*Mouse testes*<br>1 µM:<br>• No change in the total number of germ cells per testis<br>• No change in the apoptotic rate of germ cells<br>• No change in T secretion	(Angenard et al., 2010)

Disease Models & Mechanisms • Supplementary information

		Mouse ovaries<br>1 µM:<br>• Decreased germ cell number after days 3 and 10<br>• Increased percentage of apoptotic germ cells after days 3 and 10<br>10 µM:<br>• Extinguished germ cell population after day 3	
Human	Primary human testis and ovary tissue cultures exposed *ex vivo* to 0.1, 1, 10, or 50 µM Cd (from $CdCl_2$) for 4 days for testes and for 8 days for ovaries	*Human testes*<br>0.1 µM:<br>• No increase in apoptotic rate of germ cells<br>• No change in T secretion<br>1 µM:<br>• Decrease in total number of germ cells per testis<br>• Increased apoptotic rate of germ cells<br>• No change in proliferation rate<br>• No change in T secretion<br>10 µM:<br>• Increased apoptotic rate of germ cells<br>• No change in T secretion<br>*Human ovaries*<br>0.1 µM:<br>• No change in germ cell density<br>• No change in percentage in apoptotic germ cells<br>• No change in proliferation rate of germ cells<br>1 µM Cd:<br>• Decreased germ cell density<br>• Increased percentage of apoptotic germ cells<br>• No change in proliferation rate of germ cells<br>10 µM Cd:<br>• Decreased germ cell density<br>• Increased percentage of apoptotic germ cells<br>• No change in proliferation rate of germ cells<br>50 µM Cd:<br>• Decreased germ cell density	

Disease Models & Mechanisms • Supplementary information

Table S11. Key findings related to liver development and disease from experimental animal models of early life Cd exposure

Species	Exposure model	Key Findings	Source
Sprague-Dawley rats	Daily maternal oral gavage of 25 mg/kg/day Cd from GD6-18	Time course from GD15-PND56:<br>• Reduced hepatic activity of fructose metabolism enzyme sorbitol dehydrogenase<br>• No difference in hepatic lactate dehydrogenase or glucose-6-phosphate dehydrogenase activity	(Stewart et al., 1984)
Wistar rats	Daily maternal SQ injection of 0.49 mg/kg Cd as $CdCl_2$ starting at conception	PND16-20:<br>• No difference in fetal liver Zn<br>• No difference in fetal liver glycogen	(Hazelhoff Roelfzema et al., 1989)
Wistar rats	60 ppm Cd as $CdCl_2$ in maternal drinking water during GD1-20	e20:<br>• Decreased fetal liver Zn	(Barański, 1986)
Wistar rats	50 or 500 mg/kg/day $CdCl_2$ in maternal drinking water for 3 weeks before mating and during gestation and lactation	PND21, 50 and 500 mg/kg/day:<br>• No difference in plasma aspartate aminotransferase (AST) or alanine aminotransferase (ALT)<br>• Dose-responsive hepatic Cd accumulation<br>PND21, 50 mg/kg/day:<br>• Impaired glucose tolerance<br>• Elevated plasma non-esterified fatty acids (NEFA), indicitive of insulin resistance<br>PND26, 50 and 500 mg/kg/day:<br>• No difference in glucose tolerance and plasma NEFA<br>• Increased plasma adiponectin<br>PND26, 500 mg/kg/day:<br>• Reduced plasma C peptide, indicative of diabetes<br>PND60, 500 mg/kg/day:<br>• Elevated plasma C peptide, indicative of insulin resistance<br>• No difference in glucose tolerance, plasma NEFA or plasma adiponectin	(Jacquet et al., 2019)
Wistar rats	10 ppm Cd as CdCl2 in maternal drinking water from weaning until mating, then 50 ppm Cd during gestation until GD20	GD20:<br>In females:<br>• Increased hepatic GC receptor (GR) gene and protein abundance<br>• Hypomethylation in the hepatic GR promoter<br>• Decreased hepatic DNA methyltransferase 3a (*Dnmt3a*) gene expression<br>In males:<br>• Decreased hepatic GR gene and protein abundance<br>• Hypermethylation in the hepatic GR promoter<br>• Increased hepatic *Dnmt3a* gene expression	(Castillo et al., 2012)
Charles Foster rats	Daily maternal SQ injection of 0.05 mg/kg/day CdAc for 5-7 days before mating, and during gestation and lactation until PND21	PND21:<br>• Decreased hepatic DNA<br>• Decreased hepatic glycogen<br>• Decreased hepatic Zn	(Pillai and Gupta, 2005)
Wistar rats	Daily maternal SQ injection of 0.49 mg/kg/day $CdCl_2$ during gestation	GD15 and GD 20:<br>• No difference in fetal hepatic glycogen	(Yoruk et al., 2003)
Sprague-Dawley rats	25, 50 or 100 µg/mL Cd as $CdCl_2$ in maternal	GD21; 25, 50, and 100 µg/mL:<br>• Dose-responsive reduced fetal hepatic Zn concentrations	(Sasser et al., 1985)

	drinking water from GD0-21	• No difference in etal hepatic MT	
Wistar rats	Daily Maternal oral gavage of 1 or 10 mg/kg $CdCl_2$ from GD9-19	GD19, 1 and 10 mg/kg:<br>• Decreased liver weight<br>GD19, 10 mg/kg only:<br>• Decreased hepatic Zn, Fe, and Cu<br>• Hepatic Cd accumulation	(Kuriwaki et al., 2005)
Sprague-Dawley	0, 5, 50, or 100 ppm Cd as $CdCl_2$ in maternal drinking water from GD6-20	GD20, 50 and 100 ppm:<br>• Decreased Zn concentrations in the liver	(Sorell and Graziano, 1990)
Wistar rats	Daily maternal SQ injection of 0.49 mg/kg Cd as $CdCl_2$ starting at conception	GD16-20<br>• No change in fetal liver glycogen	(Roelfzema et al., 1987)
Wistar rats	50 mg/L Cd as $CdCl_2$ in maternal drinking water during gestation and lactation with or without 60 mg/L Zn as $ZnCl_2$	PND21:<br>• Decreased hepatic insulin-like growth factor-I (IGF-I) in males	(Mimouna et al., 2018)
Wistar rats	50 ppm Cd as $CdCl_2$ in maternal drinking water during gestation until GD20	GD20:<br>• Decreased fetal hepatic Zn, Fe, MT, and thionein-Zn<br>• Decreased hepatic nuclear and cytoplasmic Zn content<br>• Decreased hepatic microsomal Fe content	(Sowa and Steibert, 1985)
Wistar rats	50 mg/L Cd from $CdCl_2$ in maternal drinking water before and during gestation and during lactation	PND0, PND11 and PND21:<br>• Decreased hepatic Fe<br>PND21:<br>• Decreased hepatic Zn<br>PND49:<br>• Increased hepatic Zn in females<br>Hepatic Cd was detected in exposed offspring at all timepoints	(Mikolić et al., 2016)
Wistar mice	30 or 75 ppm Cd in maternal and paternal drinking water for two months before mating, and during gestation and lactation.	PND7, 75 ppm:<br>• Increased hepatic lipid peroxidation	(Xu et al., 1993)
CD-1 mice	500 ppb CdCl2 in maternal drinking water for two weeks before mating until PND10	PND42, females only:<br>• Increased dyslipidemia and hepatic lipid deposition<br>• Altered expression of MASLD-related genes<br>• Impaired glucose tolerance and insulin tolerance<br>PND90 and PND120, females only:<br>• Increased incidence of preneoplastic hepatic lesions	(Jackson et al., 2020)
C57BL/6J mice	5 ppm Cd from $CdCl_2$ in maternal and paternal drinking water for 16 weeks before mating and until PND21	29 weeks old:<br>• Increased susceptibility to HCC induction in males<br>• Impaired insulin tolerance in females	(Men et al., 2021)
C57BL/6J x CAST/EiJ hybrids	50 ppm CdCl2 in maternal drinking water for 5 weeks before mating and during gestation	PND0:<br>• Increased hepatic Na, Mg, and Mn<br>• Decreased hepatic Ca, Fe, K, P, S, Zn, Co, Cu, Mo, and Se	(Hudson et al., 2019)
C57BL/6J mice	Daily maternal oral gavage of 5 or 10 mg/kg Cd from $CdCl_2$ from GD1-18	GD19, 5 and 10 mg/kg:<br>• Decreased hepatic expression of polyubiquitin gene *Ubc*<br>GD19, 10 mg/kg only:<br>• Accumulation of hepatic polyubiquitinated protein	(Kurita et al., 2018)

		• Decreased hepatic monoubiquitin protein	
ICR mice	50 or 150 mg/L CdCl2 in maternal drinking water from GD8-17, with or without 500 mg/kg NAC supplementation	PND35, 50 and 150 mg/L:<br>• Decrease liver/fetal weight ratio<br>• Increased hepatic oxidative stress protein HO-1<br>• Increased hepatic expression of gluconeogenesis gene *G6pc* and proteins p-Creb and Pcg-1α<br>PND35, 150 mg/L only:<br>• Reduced fetal liver weight<br>• Increased hepatic oxidative stress proteins Nox2, Nox4, and Sod2<br>• Increased hepatic SOD activity<br>• Reduced hepatic GSH<br>• Hyperglycemia in males<br>• Increased hepatic expression of gluconeogenesis genes *Pcg-1α, Pepck,* and *Fbp1* and proteins Pepck and G6pc<br>PND98, 150 mg/L only:<br>• Increased expression of gluconeogenesis proteins p-Creb, Pgc-1, and G6pc in males<br>• Impaired glucose tolerance in males<br>• No observed insulin resistance in males<br>• No effect on serum ALT<br>With NAD supplementation:<br>• Glucose tolerance and oxidative stress parameters largely returned to normal	(Yi et al., 2021)
C57BL/6J mice	Daily maternal oral gavage with 5 mg/kg Cd as from GD1-18	GD19:<br>• 2.0-fold or more increase in 1,669 genes<br>• 0.5-fold or less decrease in 194 genes<br>• Altered pathways included cell cycle and cell proliferation, apoptosis, cell growth and differentiation, cellular defense, metabolism, transport, transcription, signal transduction, metal homeostasis, and ubiquitin protease system	(Kurita et al., 2016)
C57BL/6J mouse	Paternal IP injection with 1.0 mg/kg $CdCl_2$ every other day for 5 weeks	PND112<br>• Increased edema, number of mitochondria, and ruptured mitochondrial membranes in hepatic cells<br>Increased immunofluorescence and protein abundance of mitochondrial and lysosome markers Vdac1/2 and Lamp2 in hepatic cells	(Zeng et al., 2022)
CD-1 mouse	0.5 mg/L $CdCl_2$ in maternal drinking water for 2 weeks prior to mating until PND10	GD18<br>• Increased Cd and Zn concentrations, decreased Fe concentration, a no change in Mn concentration in female livers<br>• Decreased Fe concentration and no change in Cd, Zn, and Mn concentrations in male liver<br>• Decreased *Mt1* mRNA abundance in female but not male livers<br>• No change in *Mt2* and *Mt3* mRNA abundance in either male or female livers<br>PND1<br>• Increased *Slc39a4* and *Slc25a16* mRNA abundance but decreased *Slc39a6* mRNA abundance in female livers<br>PND21<br>• Increased Cd concentration, decreased Zn and Mn concentrations, and no change in Fe concentration in female livers<br>• Metal content was below the limit of detection in male livers<br>• Increased *Slc39a4*, *Slc30a10*, and *Slc25a16* mRNA abundance but decreased *Slc39a14* mRNA abundance in female livers	(Jackson et al., 2022)

Disease Models & Mechanisms • Supplementary information

		PND42<br>• Increased Cd concentration but decreased Zn, Fe, and Mn concentrations in female livers<br>• Increased Cd concentration but no change in Zn, Fe, and Mn concentrations in male livers<br>• Increased *Slc30a6*, *Slc30a10*, *Slc39a3*, *Slc39a6*, *Slc39a8*, and *Slc39a13* mRNA abundance but decreased *Slc39a2*, *Slc39a4*, *Slc39a14*, *Mt1*, and *Mt3* mRNA abundance in female livers<br>PND90<br>• Increased Cd concentration but decreased Zn, Fe, and Mn concentrations in female livers<br>• Decreased Fe concentration and no change in Cd, Zn, and Mn concentrations in male livers<br>PND120<br>• Increased Cd concentration but decreased Zn, Fe, and Mn concentrations in female livers<br>Decreased Fe concentration and no change in Cd, Zn, and Mn concentrations in male livers	
CD-1 mouse	50 or 150 mg/L $CdCl_2$ in maternal drinking water from GD8-17, with or without daily 15 µg/kg E2 (E2) IP injections from GD8-17 (only for 150 mg/L group)	GD18, 50 mg/L<br>• Decreased fetal liver weight in males<br>• Decreased Ki67 immunofluorescence in male livers<br>• Decreased Pcna protein abundance but no change in CyclinD1 in male livers<br>• Decreased sinusoidal area in male livers<br>• Decreased *Dll4* mRNA abundance in male livers<br>• Decreased Vegf-a and Notch3 protein abundance but no change in Dll4 in male livers<br>• No change in E2 levels in male livers<br>• Decreased Erα protein abundance but no change in Cyp17a1, 17β-hsd, and Erβ in male livers<br>GD18, 150 mg/L<br>• Decreased fetal liver weight and liver to body weight percentage in males<br>• Decreased Ki67 immunofluorescence in male livers<br>• Decreased Pcna and CyclinD1 protein abundance in male livers<br>• Decreased sinusoidal area in male livers<br>• Decreased *Notch3* and *Dll4* mRNA abundance in male livers<br>• Decreased Vegf-a, Dll4, and Notch3 protein abundance in male livers<br>• Decreased E2 levels in male livers<br>• Decreased Erα protein abundance but no change in Cyp17a1, 17β-hsd, and Erβ in male liver<br>GD18, 150 mg/L with E2<br>• Rescued fetal liver weight and liver to body weight percentage in males<br>• Improved Ki67 immunofluorescence in male livers<br>• Improved Pcna, CyclinD1, and Erα protein abundance in male livers<br>• Improved sinusoidal area in male liver<br>• Alleviated Vegf-a, Dll4, and Notch3 protein abundance deficiency in male livers	(Fu et al., 2023)
C57BL/6J mouse	Paternal IP injection with 1.0 mg/kg $CdCl_2$ every other day for 5 weeks	PND112<br>• Impaired glucose tolerance in males and females	(Zeng et al., 2023)

Disease Models & Mechanisms • Supplementary information

		• Increased random serum glucose and fasting serum insulin levels in males and females<br>• Increased serum total cholesterol (TC) and low density lipids (LDL), decreased triglycerides (TG), and no change in high density lipids (HDL) in females<br>• Increased serum TC, TG, and LDL but no change in HDL in males<br>• Increased lipid deposition in livers of males and females<br>• Reduced unsaturated fatty acid levels in livers of males<br>• Transcriptomics revealed enrichment for terms linked to atty acid metabolism, long-chain fatty acid metabolism, unsaturated fatty acid metabolism, and arachidonic acid metabolism in livers of male mice<br>• Gene set enrichment analysis revealed significant downregulation of arachidonic acid metabolism, AMPK signaling pathway, PPAR signaling pathway, and adipocytokine signaling pathway livers of male mice<br>• Increased *Acly*, *Acaca*, *Fasn* and *Scd1* mRNA abundance and increased Fasn protein abundance in livers of males<br>• Decreased *Acsl1* and *Cpt1a* mRNA and protein abundance in livers of males<br>• Decreased *Ppard*, *Cd36*, *Cyp4a14*, *Cyp4a10*, and *Pck1* mRNA abundance and Cd36, Pck1, and Ppard protein abundance in livers of males	
C57BL/6 mouse	10 or 100 mg/L $CdCl_2$ in paternal drinking for 10 weeks	GD18, 10 mg/L<br>• No change in liver weight or liver to body weight percentage in males and females<br>GD18, 100 mg/L<br>• No change in liver weight or liver to body weight percentage in males and females<br>• Differential gene expression including pathways enriched for phospholipid metabolism in livers of females<br>• Decreased *Pitpnb*, *Atp11a*, *Plekha3*, *Scp2*, and *Apoc3* mRNA abundance in livers of females<br>• Altered hepatic lipidomic profile including multiple phospholipids in livers of females<br>• No change in hepatic lipidomic profile in livers of males<br>PND105, 10 mg/L<br>• No change in fasting blood glucose or glucose tolerance in males or females<br>PND 105, 100 mg/L<br>• Increased fasting blood glucose and impaired glucose tolerance in females<br>• No change in fasting blood glucose or glucose tolerance in males<br>PND125, 10 mg/L<br>• No change in liver weight or liver to body weight percentage in males and females<br>PND125, 100 mg/L<br>• No change in liver weights or liver to body weight percentage in males<br>• Decreased liver weight but no change in liver to body weight percentage in females<br>• Increased hepatic glucose content in livers of females<br>• Increased p-Gs protein abundance in livers of females	(Tan et al., 2023)

Disease Models & Mechanisms • Supplementary information

		• Altered hepatic lipidomic profile including phosphatidylcholine (PC), lyso PC (LPC), phosphatidylinositols (PI) and phosphatidylserines (PS) in livers of females<br>• Little to no change in lipidomic profile in livers of males<br>• 296 differentially expressed genes including pathways to glucose and lipid metabolism in livers of females<br>• Increased *Ppplr3c*, *Pfkfb1*, *Dcxr* and *Gstm2* mRNA abundance in livers of females<br>Increased Ahr protein abundance but decreased IR and pAKT in livers of females	
C57BL/6 moused	20 and 40 mg/L Cd as $CdCl_2$ in maternal drinking water from E7.5-19.5	E16.5, All doses<br>• No change in total amino acid levels in livers<br>E19.5, All doses<br>• Decreased total amino acid levels in livers	(Xu et al., 2023)
BxC hybrid mouse	1 or 50 ppm $CdCl_2$ in C57BL/6J maternal drinking water for 5 weeks prior to mating and during gestation until PND10	PND0, 1 ppm<br>• No change in liver mass in males or females<br>• No evidence of hepatic lipid accumulation in males or females<br>• No change in the expression of steatosis, inflammation, and fibrosis genes in livers of males or females<br>PND0, 50 ppm<br>• No change in liver mass in males or females<br>• No evidence of hepatic lipid accumulation in males or females<br>• No change in the expression of steatosis, inflammation, and fibrosis genes in livers of males or females<br>PND21, 1 ppm<br>• No change in liver mass in males or females<br>• No change in hepatic lipid or collagen deposition in males or females<br>• No change in the expression of steatosis, inflammation, and fibrosis genes in livers of males or females<br>PND21, 50 ppm<br>• Decreased liver mass in males but no change in females<br>• Increased hepatic lipid deposition in males and females<br>• Increased hepatic collagen deposition in females but no change in males<br>• Increased expression of steatosis, inflammation, and fibrosis genes in livers of males and females<br>• Increased expression of imprinted genes in livers of males and females<br>• No change in methylation status of Zac1 imprinted control region in livers of males or females<br>PND90, 1 ppm<br>• No change in liver mass in males or females<br>• No change in hepatic lipid deposition in males or females<br>• No change in the expression of steatosis, inflammation, and fibrosis genes in livers of males or females<br>PND90, 50 ppm<br>• Increased liver mass in males but no change in females<br>• No change in hepatic lipid deposition in males or females	(Riegl et al., 2023)

Disease Models & Mechanisms • Supplementary information

		No change in the expression of steatosis, inflammation, and fibrosis genes in livers of males or females	
Ross 306 broiler chickens	*In ovo* injection of 2, 4 or 8 μg/egg Cd as $CdCl_2$ on E4	E14, 8 μg/egg:<br>• Increase in the number and size of mitochondria<br>• Increase in the expansion of the lysosomal compartment<br>• More autophagic vacuoles in hepatocytes<br>E18; 2, 4, and 8 μg Cd/egg:<br>• Changes in the structure and size of hepatocyte mitochondria<br>• Dose-responsive increase in hepatocyte swelling<br>PND1; 2, 4, and 8 μg Cd/egg:<br>• Increase in hepatocyte mitochondrial swelling<br>• Increase in hepatocyte rough ER<br>• Extended hepatocyte lysosomal system	(Dżugan et al., 2018)
Broiler chickens	*In ovo* injection of 0.43 or 430 μM $CdCl_2$	E14, 430 μM:<br>• Sinusoidal dilation and hepatic tissue necrosis<br>• Ruptured hepatocyte cellular membranes<br>• Irregular hepatocyte chromatin condensation<br>• Damaged hepatocyte organelles	(Venter et al., 2015)

Table S12. Key finding related to kidney development and disease from experimental animal models of early life Cd exposure

Species	Exposure Model	Key Findings	Source
Wistar rats	Maternal inhalation of 1.48 mg/kg/ day Cd from a $CdCl_2$ solution from GD8-20	GD21:<br>• Markers of renal injury in amniotic fluid:<br>• Elevated albumin, OPN, VEGF, and TIMP-1<br>• Decreased creatinine<br>• No difference in clusterin, calbindin, and IFN-inducible protein 10 (IP-10)<br>• Tubular damage and precipitations in the renal pelvis<br>• Cd accumulation in fetal kidney	(Jacobo-Estrada et al., 2016)
Wistar rats	Maternal oral gavage of 0.5 mg/kg/day $CdCl_2$ during gestation	GD20-PND60:<br>• Increasing Cd accumulation over time<br>PND60:<br>• Loss of renal function indicated by decreased GFR and urinary inulin<br>• Tubular dysfunction indicated by increased ion excretion fraction<br>• Altered tubular distribution of claudin proteins (CLDN2 and CLDN5)	(Jacquillet et al., 2007)
Wistar rats	Maternal inhalation of 1.48 mg/kg/day Cd from $CdCl_2$ during GD8-20	GD21:<br>• Reduced renal *Vegf* gene expression<br>• Reduced renal DNA binding ability of HIF-1, a hypoxia-induced transcription factor<br>• No change in renal PHD1, HIF-1, or VEGF protein levels	(Jacobo-Estrada et al., 2018)
Wistar rats	50 mg/L Cd from $CdCl_2$ in maternal drinking water before and during gestation and during lactation	PND0 and PND11:<br>• Decreased renal Fe<br>PND21<br>• Decreased renal Fe<br>• Increased renal Zn<br>PND49:<br>• Increased renal Zn in females	(Mikolić et al., 2016)
Wistar rats	Maternal oral gavage of 0.5 mg/kg/day $CdCl_2$ from GD9-19	GD19:<br>• Decreased renal Na/K ratio<br>• No difference in renal Na, K, Zn, Cu, Fe, Mg, or P<br>• No renal Cd detected	(Kuriwaki et al., 2005)
Sprague-Dawley rats	Maternal IP injection of 2.5 mg/kg $CdCl_2$ on GD8, 10, 12, and 14	PND3:<br>• Decreased renal alkaline phosphatase (ALP) activity<br>PND3 and PND12:<br>• No renal Cd detected<br>• No change in MT levels	(Saillenfait et al., 1992)
Sprague-Dawley rats	Maternal IP injection of 2.0 or 2.5 mg/kg $CdCl_2$ on GD8, 10, 12, and 14	PND3, 2.0 and 2.5 mg/kg<br>• Elevated gamma glutamyl transferase (GGT)<br>• Elevated ALP<br>• Elevated *N*-acetyl-β-glucosaminidase (NAG)<br>PND3, 2.5 mg/kg only<br>• Proximal tubule dysfunction indicated by β_2-microglobulin (β_2-m)<br>PND12, 2.0 and 2.5 mg/kg<br>• All parameters returned to normal	(Saillenfait et al., 1991)
Wistar rats	50 ppm Cd from $CdCl_2$ in maternal drinking water for 5 months before mating and until GD20	GD20:<br>• No detected renal Cd<br>• No change in renal succinic dehydrogenase, NADP-dehydrogenase, Mg-activated ATPase, or acid phosphatase activity<br>• Reduced number of developed nephrons	(Steibert et al., 1984)
CD-1 mice	Maternal inhalation of 230 μg/m^3 CdO	PND10 and PND14	(Blum et al., 2015)

Disease Models & Mechanisms • Supplementary information

	nanoparticles during GD4.5-16.5	• Decreased renal kidney injury molecule (*Kim-1*) gene expression<br>• No change in renal gene expression of NGAL, another kidney injury biomarker<br>• No change in urinary creatine or Kim-1	

Disease Models & Mechanisms • Supplementary information
